# Targeting epigenetic regulators for cancer therapy: mechanisms and advances in clinical trials

**DOI:** 10.1038/s41392-019-0095-0

**Published:** 2019-12-17

**Authors:** Yuan Cheng, Cai He, Manni Wang, Xuelei Ma, Fei Mo, Shengyong Yang, Junhong Han, Xiawei Wei

**Affiliations:** Laboratory of Aging Research and Cancer Drug Target, State Key Laboratory of Biotherapy and Cancer Center, National Clinical Research Center for Geriatrics, West China Hospital, Sichuan University, Chengdu, China

**Keywords:** Drug development, Cancer epidemiology

## Abstract

Epigenetic alternations concern heritable yet reversible changes in histone or DNA modifications that regulate gene activity beyond the underlying sequence. Epigenetic dysregulation is often linked to human disease, notably cancer. With the development of various drugs targeting epigenetic regulators, epigenetic-targeted therapy has been applied in the treatment of hematological malignancies and has exhibited viable therapeutic potential for solid tumors in preclinical and clinical trials. In this review, we summarize the aberrant functions of enzymes in DNA methylation, histone acetylation and histone methylation during tumor progression and highlight the development of inhibitors of or drugs targeted at epigenetic enzymes.

## Introduction

**Fig. 1:**
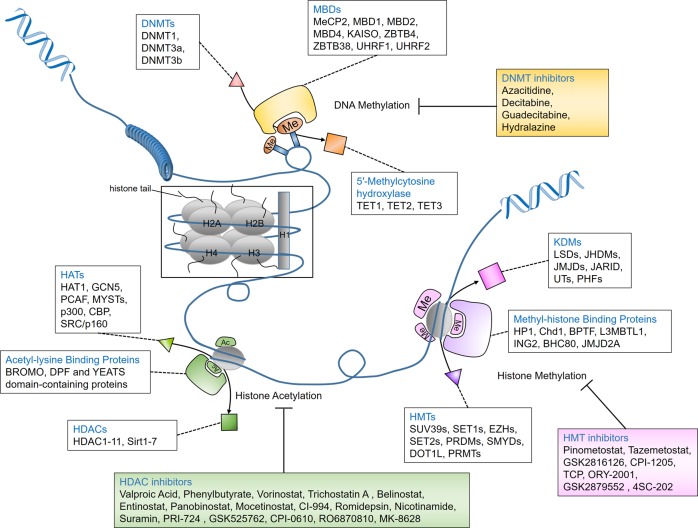
Epigenetic regulation of DNA methylation, histone acetylation, and histone methylation. Gene silencing in mammalian cells is usually caused by methylation of DNA CpG islands together with hypoacetylated and hypermethylated histones. The “writers” (DNMTs, HATs, and HMTs) and “erasers” (DNA-demethylating enzymes, HDACs, and KDMs) are enzymes responsible for transferring or removing chemical groups to or from DNA or histones; MBDs and other binding proteins are “readers” that recognize methyl-CpGs and modified histones. DNMTs, DNA methyltransferases; MBDs, methyl-CpG binding domain proteins; HATs, histone acetylases; HDACs, histone deacetylases; HMTs, histone methyltransferases; KDMs, histone-demethylating enzymes.

After the discovery of DNA and the double helix structure, classic genetics has long assumed that the sequences of DNA determine the phenotypes of cells. DNA is packaged as chromatin in cells, with nucleosomes being the fundamental repeating unit. Four core histones (H2A, H2B, H3, and H4) form an octamer and are then surrounded by a 147-base-pair (bp) segment of DNA. Nucleosomes are separated by 10–60 bp DNA. Researchers have gradually found organisms that share the same genetic information but have different phenotypes, such as somatic cells from the same individual that share a genome but function completely differently. The term epigenetics was first proposed and established in 1942 when Conrad Waddington tried to interpret the connection between genotype and phenotype.^1^ Later, Arthur Riggs and his group interpreted epigenetics as inherited differences in mitosis and meiosis, which could explain the changes in phenotypes. They were both trying to find the link between genotype and phenotype. Epigenetics is usually referred to as a genomic mechanism that reversibly influences gene expression without altering DNA sequences. Holliday assumed that epigenetics was also mitotically and/or meiotically heritable without DNA sequence change. Aberrant DNA methylation could be repaired via meiosis, but some patterns are still transmitted to offspring.^2^ This phenomenon covers a wide range of cellular activities, such as cell growth, differentiation, and disease development, and is heritable.^3^ Generally, epigenetic events involve DNA methylation, histone modification, the readout of these modifications, chromatin remodeling and the effects of noncoding RNA. The elements involved in different modification patterns can be divided into three roles, “writer,” “reader,” and “eraser”. The “writers” and “erasers” refer to enzymes that transfer or remove chemical groups to or from DNA or histones, respectively. “Readers” are proteins that can recognize the modified DNA or histones (Fig. 1). To coordinate multiple biological processes, the epigenome cooperates with other regulatory factors, such as transcription factors and noncoding RNAs, to regulate the expression or repression of the genome. Epigenetics can also be influenced by cellular signaling pathways and extracellular stimuli. These effects are temporary and yet long-standing. Given the importance of epigenetics in influencing cell functions, a better understanding of both normal and abnormal epigenetic processes can help to understand the development and potential treatment of different types of diseases, including cancer.

**Fig. 2:**
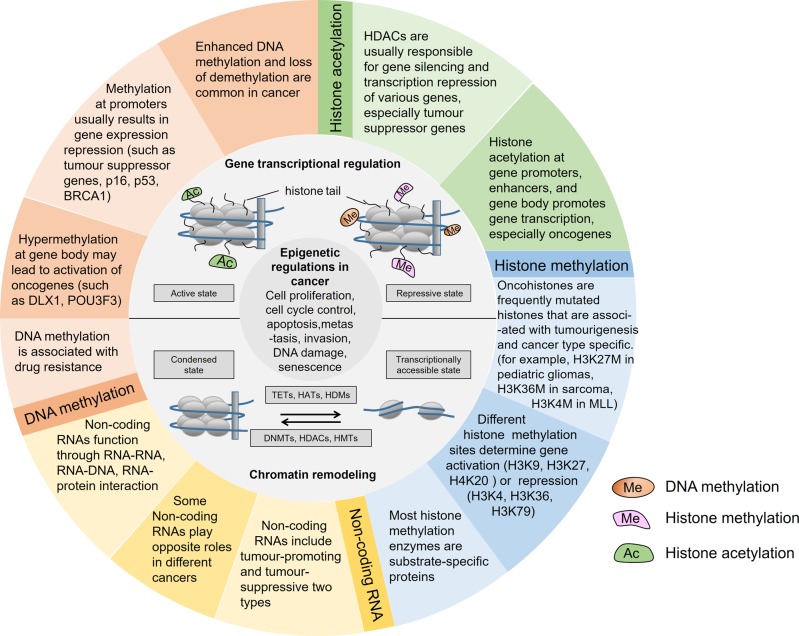
Epigenetic regulations in cancer. Alterations in epigenetic modifications in cancer regulate various cellular responses, including cell proliferation, apoptosis, invasion, and senescence. Through DNA methylation, histone modification, chromatin remodeling, and noncoding RNA regulation, epigenetics play an important role in tumorigenesis. These main aspects of epigenetics present reversible effects on gene silencing and activation via epigenetic enzymes and related proteins. DNMTs, DNA methyltransferases; TETs, ten-eleven translocation enzymes; HATs, histone acetylases; HDACs, histone deacetylases; HMTs, histone methyltransferases; HDMs, histone-demethylating enzymes. MLL, biphenotypic (mixed lineage) leukemia.

The etiology of cancer is quite complicated and involves both environmental and hereditary influences. In cancer cells, the alteration of genomic information is usually detectable. Like genome instability and mutation, epigenome dysregulation is also pervasive in cancer (Fig. 2). Some of the alterations determine cell function and are involved in oncogenic transformation.^4^ However, by reversing these mutations by drugs or gene therapy, the phenotype of cancer can revert to normal. Holliday proposed a theory that epigenetic changes are responsible for tumorigenesis. The alteration of cellular methylation status by a specific methyltransferase might explain the differences in the probability of malignant transformation.^5^ In clinical settings, we noticed that although cancer patients share the same staging and grade, they present totally different outcomes. In tumor tissues, different tumor cells show various patterns of histone modification, genome-wide or in individual genes, indicating that epigenetic heterogeneity exists at a cellular level.^6^ Likewise, using molecular biomarkers is thought to be a potential method to divide patients into different groups. It is important to note that tumorigenesis is the consequence of the combined action of multiple epigenetic events. For example, the repression of tumor suppressor genes is usually caused by methylation of DNA CpG islands together with hypoacetylated and hypermethylated histones.^7^ During gene silencing, several hallmarks of epigenetic events have been identified, including histone H3 and H4 hypoacetylation, histone H3K9 methylation, and cytosine methylation.^8,9^

Therefore, epigenetics enables us to investigate the potential mechanism underlying cancer phenotypes and provides potential therapy options. In this review, we focused and briefly expanded on three aspects of epigenetics in cancer: DNA methylation, histone acetylation and histone methylation. Finally, we summarized the current developments in epigenetic therapy for cancers.

## DNA methylation

The DNA methylation pattern in mammals follows certain rules. Germ cells usually go through a stepwise demethylation to ensure global repression and suitable gene regulation during embryonic development. After implantation, almost all CpGs experience de novo methylation except for those that are protected.^10^ Normal dynamic changes in DNA methylation and demethylation based on altered expression of enzymes have been known to be associated with aging.^11,12^ However, inappropriate methylation of DNA can result in multiple diseases, including inflammatory diseases, precancerous lesions, and cancer.^13–15^ Of note, de novo methylation of DNA in cancer serves to prevent reactivation of repressed genes rather than inducing gene repression.^16^ Because researchers have found that over 90% of genes undergoing de novo methylation in cancer are already in a repressed status in normal cells.^17^ Nevertheless, aberrant DNA methylation is thought to serve as a hallmark in cancer development by inactivating gene transcription or repressing gene transcription and affecting chromatin stability.^18^

The precise mechanism by which DNA methylation affects chromatin structure unclear, but it is known that methyl-DNA is closely associated with a closed chromatin structure, which is relatively inactive.^19^ Hypermethylation of promoters and hypomethylation of global DNA are quite common in cancer. It is widely accepted that gene promoters, especially key tumor suppressor genes, are unmethylated in normal tissues and highly methylated in cancer tissues.^20^ P16, a tumor suppressor encoded by CDKN2A, has been found to gain de novo methylation in ~20% of different primary neoplasms.^21^ Mutations in important and well-studied tumor-suppressive genes, such as P53 and BRCA1, are frequently identified in multiple cancers.^22–24^ Studies have found that the level of methylation is positively associated with tumor size. In support of this, a whole-genome methylation array analysis in breast cancer patients found significantly increased CpG methylation in FES, P2RX7, HSD17B12, and GSTM2 coincident with increasing tumor stage and size.^25^ After analysis of long-range epigenetic silencing at chromosome 2q14.2, methylation of EN1 and SCTR, the first well-studied example of coordinated epigenetic modification, was significantly increased in colorectal and prostate cancers.^26,27^ EN1 methylation has also been observed to be elevated by up to 60% in human salivary gland adenoid cystic carcinoma.^28^ Of note, only ~1% of normal samples exhibited EN1 CpG island hypermethylation.^26^ Therefore, the significant difference between cancer cells and normal cells makes EN1 a potential cancer marker in diagnosis. In human pancreatic cancer, the APC gene, encoding a regulator of cell junctions, is hypermethylated by DNMT overexpression.^29^ During an analysis of colorectal disease methylation patterns, researchers found several genes that showed significant changes between precancerous diseases and cancers, including RUNX3, NEUROG1, CACNA1G, SFRP2, IGF2 DMR0, hMLH1, and CDKN2A.^30^ In the human colon cancer cell line HCT116, hMLH1 and CDKN2A always bear genetic mutation and hypermethylation of one allele, and this leads to inactivation of key tumor suppressors.^31^ It is known that p16, p15, and pax6 are usually aberrantly methylated in bladder cancer and show enhanced methylation in cell culture.^32^. Unlike gene promoter methylation, gene body methylation usually results in increased transcriptional activity.^33^ This process often occurs in CpG-poor areas and causes a base transition from C to T.^34^ The hypermethylation of specific CpG islands in cancer tissues is informative of mutations when the gene in normal tissues is unmethylated. One representative marker is glutathione S-transferase-π (GSTP1), which is still the most common alteration in human prostate cancer.^35^ Recently, DNA methylation in cancer has generally been associated with drug resistance and predicting response to treatment.^36^ For example, MGMT (O-6-methylguanine DNA methyltransferase) hypermethylation is still the best independent predictor of response to BCNU (carmustine) and temozolomide in gliomas because hypermethylation of MGMT makes tumor cells more sensitive to treatments and is associated with regression of tumor and prolonged overall survival.^37,38^ Similarly, MGMT is also a useful predictor of response to cyclophosphamide in diffuse large B-cell lymphoma^39^ (Table 1).

**Table 1:** Key regulatory factors of DNA methylation in cancer.

Enzyme	Roles in cancer	Cancer type	Associated biological process (involved mechanism and molecules)
*DNA methyltransferases*			
DNMT1: DNMT1 is responsible for maintenance of DNA methylation and is expressed at high concentrations in dividing cells to guard existing methylated sites.			
	Promoter	AML, CML, cervical cancer, colorectal cancer, glioma, lung cancer, pancreatic cancer, gastric cancer, hepatocellular carcinoma, breast cancer, esophageal cancer, bladder cancer, prostate cancer, thyroid cancer, ovarian cancer^92–100^	Promotes EMT phenotype, cell apoptosis, cell proliferation, migration, cancer stemness, and cisplatin sensitivity (β-catenin, E-cadherin, PTEN, p18, p27, P21, P16, miR-124, miR-148a, miR-152, miR-185, miR-506), DNMT1 is also upregulated by *Helicobacter pylori* CagA
	Suppressor	Prostate cancer, cervical cancer^101,102^	Cell migration, EMT and stem cell potential
DNMT3a: DNMT3a methylates unmethylated DNA de novo and is required for maternal imprinting at different methylated regions.			
	Promoter	Cervical cancer, CML, breast cancer, gastric cancer, prostate cancer, ovarian cancer, bone cancer, testicular cancer^52,103–107^	Promotes cell proliferation and invasion. (VEGFA, Wnt/β-catenin signaling, miR-182, miR-708-5p)
	Suppressor	Lymphoma, AML, breast cancer, colorectal cancer, lung cancer^108–110^	Low level of DNMT3a is associated with the poor survival of cancer patients and promotes tumor progression but not initiation
DNMT3b: DNMT3b is also responsible for de novo methylation and is required for methylation of centromeric minor satellite repeats and CGIs in inactive X chromosomes.			
	Promoter	CML, AML, glioma, lung cancer, breast cancer, gastric cancer, colorectal cancer, prostate cancer, pancreatic cancer, bladder cancer, cervical cancer^52,94,111–113^	Promotes cell proliferation, and invasion and the chemotherapy effects of cisplatin; is associated with poor prognosis (E-cadherin, PTEN, P21, P16, miR-29b, miR-124, miR-506)
	Suppressor	AML, bladder cancer^109,114^	Downregulation of DNMT3a is associated with poor prognosis
*Methyl-CpG binding proteins*			
MeCP2	Promoter	Prostate cancer, colorectal cancer, breast cancer, gastric cancer^115,116^	Promotes cell proliferation, invasion, metastasis, apoptosis, cell cycle arrest in the G0/G1 phase, chemotherapy effects, regulation of estrogen receptor status, involves the MEK1/2-ERK1/2 signaling pathway (miR-638, miR-212)
	Suppressor	Pancreatic cancer^117^	Decreased expression of MeCP2 contributes to cancer development
MBD1	Promoter	Pancreatic cancer, prostate cancer^118,119^	Promotes cell EMT, proliferation, invasion, and metastasis and the chemoradioresistance of cancer cells and induces an antioxidant response (E-cadherin)
MBD2	Promoter	Lung cancer, colon cancer, breast cancer, prostate cancer^94,120–122^	Promotes cell invasion and metastasis (p14)
MBD4	Promoter	Colon cancer, breast cancer^123,124^	Causes dominant negative impairment of DNA repair
KAISO (ZBTB33)	Promoter	Colon cancer, cervical cancer, prostate cancer, ovarian cancer, lung cancer, breast cancer, and chronic myeloid leukemia^125–128^	Silencing of tumor suppressor genes, EMT, apoptosis, migration and invasion (Wnt/β-catenin, TGFβ, EGFR, Notch, miR-4262, miR-31)
ZBTB4	Suppressor	Breast cancer, Ewing sarcoma, prostate cancer, bladder cancer^77,129–131^	Promotes cell growth and apoptosis and controls the cellular response to p53 activation, promoting long-term cell survival (miR-17-92/106b-25
ZBTB38	Promoter	Bladder cancer^132^	Promotes cell migration and invasion (Wnt/β‑catenin pathway)
UHRF1	Promoter	Hepatocellular carcinoma, bladder cancer, renal cell carcinoma, lung cancer, retinoblastoma, intrahepatic cholangiocarcinoma, colon cancer, pancreatic cancer, gastric cancer, prostate cancer, melanoma, hepatoblastoma, esophageal squamous cell carcinoma, cervical cancer, breast cancer, thyroid cancer^133–138^	Promotes cell proliferation, EMT, and viability, increases hypoxia inducible factor (HIF)1α, CSCs, taxane resistance correlates with poor pathological characteristics, human papillomavirus (HPV) contributes to overexpression of UHRF1 (miR-101, miR-124, PI3K/Akt signaling pathway, MEK/ERK pathway)
UHRF2	Promoter	Intrahepatic cholangiocarcinoma, hepatocellular carcinoma, colon cancer^139,140^	Promotes cell migration and invasion, and is associated with lower disease-free survival
	suppressor	Colon cancer, lung cancer, esophageal carcinoma^141,142^	Low level of UHRF2 is associated with shorter overall survival, vascular invasion and poor prognosis
*DNA demethylases*			
TET1: TET1 is highly expressed in mouse embryonic stem cells, the inner cell mass of blastocysts, and developing PGCs.			
	Promoter	MLL-rearranged leukemia, AML, breast cancer, ovarian cancer, lung cancer, renal cancer^143–147^	TET1-MLL fusion, cell migration, anchorage-independent growth, cancer stemness, and tumorigenicity, prevention of senescence via loss of p53, associated with a worse overall survival and sensitivity to drugs (PI3K-mTOR pathway)
	Suppressor	Hematopoietic malignancy, hepatocellular carcinoma, prostate cancer, colon cancer, gastric cancer, breast cancer, nasopharyngeal carcinoma cells, ovarian cancer^90,148,149^	Promotes EMT and increases cancer cell growth, migration, and invasion (miR-21-5p, Wnt/β-catenin signaling pathway, AKT and FAK pathways)
TET2/TET3: TET2 and TET3 are present in multiple mouse adult tissues, whereas only TET3 is present in mouse oocytes and one-cell zygotes			
TET2	Suppressor	MDS, AML, CML, prostate cancer, gastric cancer, breast cancer, colorectal cancer, ovarian cancer, hepatocellular carcinoma, leukemia^87,150–153^	Promotes cell proliferation, colony formation, metastasis, is associated with reduced patient survival, pathologic stage, tumor grading, lymph node metastasis, and vascular thrombosis (caspase-4, ET2/E-cadherin/β-catenin regulatory loop)
TET3	Promoter	Renal cell carcinoma^154^	Acts as an independent predictor of poor outcome
	Suppressor	Head and neck cancer, ovarian cancer, breast cancer^155,156^	Is associated with EMT, overall survival, disease-free survival (miR-30d)

*AML* acute myeloid leukemia, *CML* chronic myeloid leukemia, *EMT* epithelial-mesenchymal transition, *VEGFR* vascular endothelial growth factor receptor

### DNA methyltransferases (DNMTs)

DNA methylation is a covalent modification of DNA and is one of the best-studied epigenetic markers. It plays an important role in normal cell physiology in a programmed manner. The best-known type of DNA methylation is methylation of cytosine (C) at the 5th position of its carbon ring (5-mC), especially at a C followed by a guanine (G), so-called CpG sites. Non-CpG methylation, such as methylation of CpA (adenine) and CpT (thymine), is not common and usually has restricted expression in mammals.^40^ CpG islands traverse ~60% of human promoters, and methylation at these sites results in obvious transcriptional regression.^41^ Meanwhile, among the ~28 million CpGs in the human genome in somatic cells, 60–80% are methylated in a symmetric manner and are frequently found in promoter regions.^42,43^ The process of DNA methylation is regulated by the DNA methyltransferase (DNMT) family via the transfer of a methyl group from S-adenosyl-L-methionine (SAM) to cytosine.^44^ There are five members of the DNMT family: DNMT1, DNMT2, DNMT3a, DNMT3b, and DNMT3L. DNMT1 is responsible for the maintenance of methyl-DNA, recognizes hemimethylated DNA strands and regenerates the fully methylated DNA state of DNA during cell division.^45^ In a recent study, DNMT1 with Stella, a factor essential for female fertility, was responsible for the establishment of the oocyte methylome during early embryo development.^46^ DNMT3a and DNMT3b are regarded as de novo methylation enzymes that target unmethylated CpG dinucleotides and establish new DNA methylation patterns, but they have nonoverlapping functions during different developmental stages.^47,48^ DNMT2 and DNMT3L are not regarded as catalytically active DNA methyltransferases. DNMT2 functions as an RNA methyltransferase, while DNMT3L contains a truncated inactive catalytic domain and acts as an accessory partner to stimulate the de novo methylation activity of DNMT3A. The DNA methyltransferase-like protein DNMT3L can modulate DNMT3a activity as a stimulatory factor.^49^

During aberrant DNA methylation, DNMTs play an important role. Compared with DNMT1 and DNMT3a, DNMT3b was significantly overexpressed in tumor tissues.^50^ Overexpression of DNMT1, DNMT3a, and DNMT3b has been observed in multiple cancers, including AML, CML, glioma, and breast, gastric, colorectal, hepatocellular, pancreatic, prostate, and lung cancers. In cervical cancer patients, DNMT1 was expressed in more than 70% of cancer cells, whereas only 16% of normal cells expressed DNMT1. The higher level of DNMT1 expression was also associated with worse prognosis.^51^ The expression of DNMT1, DNMT3a, and DNMT3b has been observed to be elevated in acute myeloid leukemia (AML) and various solid cancers. These three methyltransferases do not show significant changes in the chronic phase of chronic myeloid leukemia (CML), but they are significantly increased during progression to the acute phase in CML.^52,53^ Notably, downregulation of DNMTs can also lead to tumorigenesis (Table 1).

### Methyl-CpG recognition proteins

How DNA methylation leads to gene repression has been considered in many studies. Several hypotheses have been proposed. Three methyl-CpG binding domain protein (MeCP) families can read the established methylated DNA sequences and in turn recruit histone deacetylases, a group of enzymes responsible for repressive epigenetic modifications, to inhibit gene expression and maintain genome integrity.^10,54^ The first group is methyl-CpG binding domain (MBD) proteins, including MeCP2, MBD1, MBD2, and MBD4. MeCP1 is a complex containing MBD2, the histone deacetylase (HDAC) proteins HDAC1 and HDAC2, and the RbAp46 and RbAp48 proteins (also known as RBBP7 and RBBP4).^55^ MBD3 is unlike the other four family members and is not capable of binding to methylated DNA but instead binds to hydroxymethylated DNA.^56^ The zinc-finger and BTB domain-containing protein family is the second group and comprises three structurally different proteins, KAISO (ZBTB33), ZBTB4 and ZBTB38, which bind to methylated DNA via zinc-finger motifs. The third family includes two ubiquitin-like proteins with PHD and RING finger domains, UHRF1 and UHRF2, which recognize 5-mC via RING finger-associated (SRA) domains. On the other hand, methylation of DNA can also be a barrier for certain transcription factors to bind to promoter sites such as *AP-2*, *c-Myc*, *CREB/ATF*, *E2F*, and *NF-kB*.^13^

As for methyl-group binding proteins, many studies have investigated their roles in various cancers, but the mechanism underlying these alterations remains unclear. MBD proteins cooperate with other proteins to regulate gene transcription.^57,58^ However, the role of MBD1 and MBD2 has not been identified in human lung or colon cancer, with only limited mutations being detected.^59^ Furthermore, loss of MBD1 did not show any carcinogenic effect in *MBD−/*− mice.^60^ Compared with MBD1, MBD2 shows more effect on tumorigenesis. Deficiency of MBD2 strongly suppresses intestinal tumorigenesis in *APC*^*Min*^-background mice.^61^ A possible reason is that many important signaling pathways are downregulated in colorectal cancer, and loss of MBD2 leads to reexpression of these genes.^62^ Meanwhile, inhibition of MBD2 shows promising effects on suppression of the tumorigenesis of human lung cancer and colon cancer.^63^ Although MBD3 does not directly bind to methylated DNA, it regulates the methylation process via interactions with other proteins, such as MBD2 and HDAC. For example, application of an HDAC inhibitor in lung cancer cells upregulated p21 (also known as CDKN1A) and downregulated ErbB2, leading to inhibition of cancer cell growth. Silencing of MBD3 blocked the effects of an HDAC inhibitor.^64^ MBD3 and MBD2 form a complex, nucleosome remodeling and deacetylase (NuRD), which interacts with histone-demethylating enzymes to regulate gene expression in cancer.^65^ Mutation of MBD4 has been found in colorectal cancer, endometrial carcinoma and pancreatic cancer.^66^ Furthermore, this mutation unexpectedly affects the stability of the whole genome, not only CpG sites.^67^ Knockout of MBD4 indeed increased tumorigenesis in *APC*^*Min*^-background mice, which makes MBD4 a tumor suppressor.^68^ MBD4 is important in DNA damage repair, given the interaction between MBD4 and MMR.^69^ In contrast, the expression of MeCP2 and the UHRF family tends to promote tumor growth.^70–74^ In the KAISO family, KAISO directly binds to p120^ctn^, a protein with an alternative location in some cancer cells, and they together regulate cell adhesion and motility.^75,76^ However, deficiency of ZBTB4 contributes to tumorigenesis^77^ (Table 1).

### DNA-demethylating enzymes

DNA methylation is a stable and highly conserved epigenetic modification of DNA in many organisms.^78^ However, loss of 5-mC and DNA demethylation have been identified in different biologic processes. For example, DNA demethylation is important for primordial germ cells (PGCs) to gain pluripotent ability.^79,80^ DNA demethylation is actively regulated by the TET protein family (ten-eleven translocation enzymes, TET1-3) via the removal of a methyl group from 5-mC. These three proteins differ from each other in terms of expression depending on the developmental stage and cell type.^18^ TETs oxidize 5-mC in an iterative manner and catalyze the conversion of 5-mC to 5-hydroxymethylcytosine (5-hmC), which is a key intermediate in the demethylation process.^81^ 5-hmC, as a relatively stable intermediate substrate, is less prone to further oxidation by TET proteins than 5-mC.^82^ However, overexpression of only TET1 and TET2 can cause a global decrease of 5-mC.^18^ Stepwise oxidation of 5-hmC by TET proteins can yield two products: 5-formylcytosine (5-fC) and 5-carboxylcytosine (5-caC).^83^ These two molecules can be excised by thymine-DNA glycosylase (TDG) and eventually be repaired to unmodified C.^84^ DNA demethylation or restoration of the unmodified cytosine can also occur passively through replication-dependent dilution of 5-mC.

Disruption of normal DNA demethylation is thought to be associated with oncogenesis. TET proteins were initially associated with leukemia. Researchers have found that in a small number of AML patients, TET1 is fused to MLL via the chromosome translocation t(10;11)(q22;q23).^85^ Further studies found that TET2 was more widely expressed in different tissues than TET1 and TET3. Analyses revealed that mutation or deficiency of TET2 occurred in ~15% of patients with myeloid cancers, including myelodysplastic syndrome (MDS), myeloproliferative disorders, and AML.^86^ In patients with CML, mutation of TET2 has been detected in ~50% of patients.^87^ Although TET2 mutations have been found in several myeloid malignancies, their prognostic effect remains controversial. Based on the phenomenon that mutation of TET2 was elevated in patients whose disease transformed from chronic myeloid malignancy to AML, researchers considered that TET2 loss was important for cells to regain the ability to self-renew.^88^ The role of TET proteins has also been investigated in several solid tumors. Compared with surrounding normal tissues, 5-hmC is significantly reduced in human breast, liver, lung, pancreatic, and prostate cancers with reduced expression of TET family proteins.^89^ Deficiency of TET1 in prostate and breast cancer is associated with tumor cell invasion and breast xenograft tumor formation via the inhibition of the methylation of metalloproteinase (TIMP) family proteins 2 and 3.^90^ Loss of 5-hmC is an epigenetic hallmark of melanoma, and thus, introducing TET2 into melanoma cells results in suppression of tumor growth and increased survival in an animal model^91^ (Table 1).

## Histone modification

Histone modification can occur to the flexible tails as well as the core domain of histones, including those sites that are buried by DNA. In particular, the flexible histone tails are enriched with basic Lys/Arg and hydroxyl group-containing Ser/Thr/Tyr residues, thereby being hotspots for hallmark histone modifications. The tails extend from the surface of the nucleosome and are readily modulated by covalent posttranslational modification (PTM). PTMs modify histones by adding or removing chemical groups and regulate many biological processes via the activation or inactivation of genes. These processes mainly include acetylation and methylation of lysines (K) and arginines (R), phosphorylation of serines (S) and threonines (T), ubiquitylation, and sumoylation of lysines. In addition to those mentioned and discussed above, histone modifications also include citrullination, ADP-ribosylation, deamination, formylation, O-GlcNAcylation, propionylation, butyrylation, crotonylation, and proline isomerization at over 60 amino acid residues.^157,158^ In addition to conventional PTMs, novel PTM sites are also found outside of the N-terminal tails.

Histone modifications at certain sites, such as promoters and enhancers, are thought to be largely invariant, whereas a small number of these sites remain dynamic. H3K4me1 and H3K27ac, two dynamic modifications, were identified to activate enhancers and regulate gene expression.^159^ H3K9ac and H3K9me3 are two common modifications at promoters.^160,161^ Appropriate histone modifications are important in gene expression and human biology; otherwise, alterations in PTMs may be associated with tumorigenesis. Analysis of cancer cells reveals that they exhibit aberrant histone modifications at individual genes or globally at the single-nuclei level.^6,162^ Understanding histone modification patterns in cancer cells can help us to predict and treat cancers. Thus far, most studies have focused on aberrant modifications within an individual site, such as H4K20me3 or H4K16ac, rather than enzymatic activity-associated abnormalities. Generally, alterations in histone modifications occur at an early stage and accumulate during tumorigenesis.^163^

### Histone acetylation (lysine)

Histone acetylation occurs at multiple lysine residues at the N-terminus via the catalysis of histone acetyltransferases (HATs), also named lysine acetyltransferases (KATs). Histone acetylation regulates the compaction state of chromatin via multiple mechanisms, such as neutralizing the basic charge at unmodified lysine residues, and is associated with active transcription, especially at gene promoters and enhancers and the gene body; it also facilitates the recruitment of coregulators and RNA polymerase complexes to the locus.^157,164^ To date, HATs and histone deacetylases (HDACs) are the two of the best characterized groups of enzymes involved in histone PTMs. HATs transfer the acetyl groups from acetyl-CoA cofactors to lysine residues at histones, whereas the role of HDACs is the opposite, which makes histone acetylation a highly reversible process.

#### Histone acetyltransferases

HATs are predominantly located in the nucleus, but multiple lines of evidence have shown lysine acetylation in the cytoplasm, and their acetylation is associated with key cellular events.^165^ In addition, lysine acetylation found outside histones reminds us of the role of HATs in nonhistone PTMs.^166^ The first HAT was identified in yeast, and was named HAT1,^167^ and was then isolated from tetrahymena as HAT A by Allis and coworkers.^168^ In humans, HATs can be roughly divided into three groups: general control nondepressible 5 (GCN5)-related N-acetyl transferase (GNAT) (based on the protein Gcn5 found in yeast; including GCN5 and PCAF), MYST (based on the protein MOZ; including MOZ, MOF, TIP60, and HBO1), and p300/cAMP-responsive element-binding protein (CBP).^169^ Other HATs, including nuclear receptors and transcription factors, such as SRC1, MGEA5, ATF-2, and CLOCK, also harbor the ability to acetylate histones. Notably, a number of acetyltransferases also perform protein acetylation outside histones, such as TFIIB, MCM3AP, ESCO, and ARD1.^170^ Knockout of CBP/p300 is lethal for early embryonic mouse models.^171,172^ The acetyl group transfer strategies for each HAT subfamily are different. For the GCN5 and PCAF family, the protein crystal structure shows a conserved glutamate in the active site. Blockade of this amino leads to a significantly decreased acetylation function.^173,174^ Similarly, there is also a conserved glutamate plus a cysteine residue located at active sites of MYST family proteins.^175^ Unlike the other two families, the p300/CBP HAT subfamily has two other potential conserved residues, a tyrosine and a tryptophan.^176^ Generally, their catalytic mechanisms of acetyl group transfer can be divided into two groups. The GNAT family depends on a sequential ordered mechanism, whereas the members of the MYST family use a so-called ping-pong (i.e., double displacement) catalytic mechanism, which means that the acetyl groups are first transferred to a cysteine residue and then transferred to a lysine residue.^177^ In addition to differences in the acetyl transfer mechanism, HAT subfamilies, even different proteins in the same family, also have remarkable diversity in targeting sites.

Appropriate acetylation within cells is important since upregulation or downregulation of HATs is associated with tumorigenesis or poor prognosis.^162,178^ Compared with solid tumors, the association between histone modifications and cancer has been widely investigated in hematological malignancies. Germline mutation of CBP results in Rubinstein-Taybi syndrome along with an increased predisposition to childhood malignancies. Meanwhile, loss of another family member, p300, has also been associated with hematological malignancies.^179,180^ Therefore, both CBP and p300 seem to function as tumor suppressors. During cancer development, the expression of HAT genes can be disrupted by chromosomal translocations, although these are rare events. Generation of the fused protein CBP-MOZ is the result of the t(8,16)(p11,p13) translocation in AML.^181^ Translocation of t(10;16)(q22;p13) leads to the CBP-MORF chimera.^182^ Similarly, p300-MOZ, MLL-CBP, and MLL-p300 (MLL, mixed lineage leukemia) have also been identified in hematological malignancies.^183–185^ Generally, chromosomal rearrangements involving CBP are more common than those involving p300. Researchers have also investigated solid tumors, which are less mutated. The expression of translocated P300 in laryngeal squamous cell carcinoma (LSCC) tissue is much higher than that in adjacent normal tissue and is associated with advanced stage and poor prognosis.^178^ Missense point mutations in p300 are found in colorectal adenocarcinoma, gastric adenocarcinoma and breast cancer with quite low incidences.^186,187^ Rare inactivating mutations in CBP and PCAF have only been identified in cancer cell lines but not primary tumors.^188^ Based on these findings, we hypothesize that the differences between cell lines and primary tumors cannot be ignored. Amplified in breast cancer 1 (AIB1), also frequently called NCOA3 (nuclear receptor coactivator 3) or SRC3 (steroid receptor coactivator 3), is overexpressed in ~60% of human breast cancers, and increased levels of AIB1 are associated with tamoxifen resistance and decreased overall survival.^189^ Steroid receptor coactivator 1 (SRC1) is also associated with the chromosomal translocation t(2;2)(q35;p23), which results in *PAX3–NCOA1* gene fusion in rhabdomyosarcoma without a consistent genetic abnormality during embryonic development^190^ (Table 2).

**Table 2:** Important enzymes or proteins that regulate histone acetylation in cancer.

Enzyme	Synonym	Role in cancer	Cancer type	Associated biological process (involved mechanism and molecules)
*Histone acetylases: the writers*				
HAT1				
HAT1	/	Promoter	Pancreatic cancer, nasopharyngeal cancer, hepatocellular carcinoma, esophageal carcinoma^227–230^	Promote cell apoptosis, proliferation, differentiation and cisplatin resistance, associated with poor prognosis and upregulates PD-L1
		Suppressor	Lung cancer, osteosarcoma^231,232^	Restores Fas expression and induces cancer cell apoptosis (Ras-ERK1/2 signaling)
GANT				
GCN5L2	GCN5	Promoter	Prostate cancer, breast cancer, non-small-cell lung cancer, colorectal cancer^233–235^	Promotes cell proliferation, apoptosis, EMT, poor prognosis of patients, promotion of E2F1, cyclin D1, and cyclin E1 expression (PI3K/PTEN/Akt signaling, TGF-β/Smad signaling pathway)
PCAF	/	Suppressor	Colorectal cancer, gastric cancer, prostate cancer, breast cancer^236–238^	Decreased PCAF is associated with 5-FU resistance, poor clinical outcome (PCAF-p16-CDK4 axis, p53, miR-17)
MYST				
HTATIP	TIP60	Promoter	Liver cancer, prostate cancer^239,240^	Promotes cancer cell EMT, metastasis, radioresistance
		Suppressor	Breast cancer, lung cancer, bladder cancer, colorectal cancer^241–243^	Is associated with cell viability and invasion, and low Tip60 expression is correlated with poor overall survival and relapse-free survival
MYST1	MOF	Promoter	Prostate cancer^244^	MYST1 increases the resistance to therapeutic regimens and promotes aggressive tumor growth (androgen receptor and NF-κB)
MYST2	HBO1	Promoter	Ovarian cancer, bladder cancer, breast cancer, pancreatic cancer, leukemia^245–247^	Promotes cell proliferation, enrichment of cancer stem-like cells, gemcitabine resistance (Wnt/β-catenin signaling)
MYST3	MOZ	Promoter	Colorectal cancer, breast cancer, leukemia^248–250^	Promotes cell proliferation, activates ERα expression (multiple fusion proteins: MOZ-TIF2, MOZ-NCOA2 and MOZ-CBP)
MYST4	MORF	Promoter	Leukemia^251^	MORF-CREBBP fusion
p300/CBP				
P300	EP300, KAT3B	Promoter	Laryngeal squamous cell carcinoma, leukemia, nasopharyngeal carcinoma, hepatocellular carcinoma, cutaneous squamous cell carcinoma, head and neck squamous cell carcinoma, colorectal cancer, breast cancer, lung cancer, gastric cancer, prostate cancer, cervical cancer, pancreatic cancer^252–257^	Promotes cell proliferation, migration, invasion, EMT, and malignant transformation, is associated with advanced clinical stage, poor recurrence-free survival and overall survival, enhances ERα expression and contributes to tamoxifen resistance, castration resistance, and gemcitabine sensitivity, (p21, p27, β-catenin, MLL-p300, MOZ-p300 fusion, Smad2 and Smad3 in the TGF-β signaling pathway, p300/YY1/miR-500a-5p/HDAC2 signaling axis)
		Suppressor	Bladder cancer, colorectal cancer^258,259^	Downregulation of P300 is associated with chemosensitivity to 5-FU treatment and doxorubicin resistance
CBP	CREBBP, KAT3A	Promoter	Lung cancer, leukemia, gastric cancer, ovarian cancer, prostate cancer, hepatocellular carcinoma^256,260–262^	Is associated with drug resistance, a highly tumorigenic, cancer stem-like phenotype and enhances the activity of estrogen receptor-beta (ER-β) (CXCL8, PI3K/Akt/β-catenin/CBP axis); KAT6A-CREBBP, MOZ-CBP, MORF-CREBBP, MLL-CBP fusions in leukemia
		Suppressor	Lung cancer, prostate cancer^263,264^	Loss of CBP reduces transcription of cellular adhesion genes while driving tumorigenesis
SRC/p160				
NCOA1	SRC1	Promoter	Prostate cancer, colon cancer, breast cancer, hepatocellular carcinoma, head and neck squamous cell carcinoma^265–267^	Promotes cell invasion, proliferation, metastasis, is associated with shorter overall survival and progression-free survival (M-CSF1, miR-4443, miR-105-1)
NCOA2	TIF2	Promoter	Prostate cancer, leukemia^268,269^	Is associated with resistance to AR antagonism and bicalutamide; MOZ-TIF2 fusion in leukemia
		Suppressor	Colorectal cancer, liver cancer^270,271^	TIF2 is able to impair protumorigenic phenotypes
NCOA3	AIB1, ACTR	Promoter	Ovarian cancer, breast cancer, bladder cancer, gastric cancer, lung cancer, prostate cancer, hepatocellular carcinoma, esophageal squamous cell carcinoma, colorectal cancer, pancreatic cancer^272–275^	Promotes cell proliferation, EMT, metastasis, invasiveness and is correlated to higher estrogen receptor expression, poor PFS and OS and predicts resistance to chemoradiotherapy (AKT, E2F1, SNAI1, cyclin E, cdk2, p53, matrix metalloproteinase 2 (MMP2) and MMP9 expression); however, high AIB1 expression has been correlated to both a good response to adjuvant tamoxifen and tamoxifen resistance.
Others				
ATF-2	CREB2, CREBP1	Promoter	Pancreatic cancer, lung cancer, renal cell carcinoma, leukemia^276–278^	Promotes cell proliferation, EMT, gemcitabine sensitivity (JNK1/c-Jun and p38 MAPK/ATF-2 pathways, miR-451); however, the level of ATF-2 is a key determinant of the sensitivity to tamoxifen
TFIIIC	/	Promoter	Ovarian cancer^279^	TFIIIC is overexpressed in cancer tissues
TAF1	TAFII250	/	/	/
CLOCK	KIAA0334	Promoter	Ovarian cancer, breast cancer^280,281^	Promotes cell proliferation, migration, and invasion, is associated with drug resistance (cisplatin)
		Suppressor	Lung cancer^282^	Is associated with cancer progression and metastasis
CIITA	MHC2TA	Suppressor	Breast cancer, colorectal cancer, gastric cancer, head and neck cancer, hepatocellular carcinoma^283–285^	Regulates the expression of MHC II and HLA-DR induction
MGEA5	NCOAT	promoter	Laryngeal cancer^286^	Is associated with larger tumor size, nodal metastases, higher grade and tumor behavior (TGFBR3-MGEA5 fusion)
		Suppressor	Breast cancer^287^	MGEA5 transcript levels were significantly lower in grade II and III than in grade I tumors; associated with lymph node metastasis
CDY	/	/	/	/
*Acetyl-lysine binding protein: the readers*				
BRD and extraterminal domain (BET) proteins family				
BRD2-4, BRDt	/	Promoter	Breast cancer, prostate cancer, gastric tumors, lung cancer, ovarian carcinoma, pancreatic cancer, hematologic malignancy, Ewing sarcoma, glioblastoma, melanoma^288–291^	Is associated with cell proliferation, self-renewal, metabolism, metastasis, and expression of immune checkpoint molecules (oncogenic AR and MYC signaling, AMIGO2-PTK7 axis, Jagged1/Notch1 signaling, IKK activity)
*Histone deacetylases (HDACs): the erasers*				
HDAC Class I				
HDAC1	/	Promoter	Thyroid cancer, lung cancer, ovarian cancer, breast cancer, colorectal cancer, pancreatic cancer, esophageal cancer, gallbladder cancer, prostate cancer, gastric cancer^292–295^	Promotes cell invasion, viability, apoptosis, EMT; is associated with chemotherapy response. (CXCL8, P53, p38 MAPK, miRNA-34a)
HDAC2	/	Promoter	Pancreatic cancer, colorectal cancer, lung cancer, squamous cell carcinoma, hepatocellular carcinoma, breast cancer, prostate cancer, renal carcinoma, ovarian cancer, gastric cancer^296–300^	Promotes cell proliferation, metastasis, invasion, clonal expansion and EMT (E-cadherin, p63, mTORC1, AKT, PELP1/HDAC2/miR-200, p300/YY1/miR-500a-5p/HDAC2 axis, Sp1/HDAC2/p27 axis)
HDAC3	/	Promoter	Colorectal cancer, pancreatic cancer, breast cancer, colorectal cancer, prostate cancer, esophageal cancer, lung cancer^301–304^	Promotes cell proliferation and invasion, migration, chemosensitivity; increases PD-L1 expression (NF‑κB signaling)
HDAC8	/	Promoter	Cervical cancer, breast cancer, colon cancer^305–307^	Promotes cell migration, affects cell morphology and promotes the cell cycle (p53, HDAC8/YY1 axis)
		Suppressor	Breast cancer^308^	HDAC8 suppresses EMT (HDAC8/FOXA1 signaling)
HDAC Class II				
HDAC4	/	Promoter	Head and neck cancer, breast cancer, colorectal cancer, gastric cancer, ovarian cancer, prostate cancer^309–311^	Promotes cell viability, drug resensitization (tamoxifen, platinum) (STAT1, p21, miR-10b)
HDAC5	/	Promoter	Breast cancer, colorectal cancer, lung cancer, prostate cancer^312,313^	Promotes cell proliferation, invasion, migration and EMT; is associated with hormone therapy resistance (HDAC5-LSD1 axis, Survivin and miR-125a-5p, miR-589-5p)
HDAC6	/	Promoter	Cervical cancer, breast cancer, colorectal cancer, gastric cancer, lung cancer, prostate cancer, liver cancer, ovarian cancer^314–317^	Promotes pluripotency of CSCs, cancer cell proliferation and migration (α-tubulin, heat shock protein (HSP) 90, the NF-κB/MMP2 pathway, JNK/c-Jun pathway, miR-22, miR-221)
HDAC7	/	Promoter	Breast cancer, colorectal cancer, prostate cancer, ovarian cancer^318–320^	Is associated with cancer stem cell-specific functions, tumor growth and invasion, and therapy resistance (miR-489, miR-34a)
HDAC9	/	Promoter	Breast cancer^321^	Enhances invasive and angiogenic potential (miR-206)
		Suppressor	Lung cancer^322^	HDAC9 is downregulated in adenocarcinomas; is associated with tumor growth ability
HDAC10	/	Promoter	Ovarian cancer, lung cancer^323,324^	Promotes cells proliferation, reduced DNA repair capacity and sensitization to platinum therapy (AKT phosphorylation)
HDAC Class III: sir2-like proteins (sirtuins)				
Sirt1	/	Promoter	Breast cancer, colorectal cancer, prostate cancer, liver cancer, lung cancer, pancreatic cancer, cervical cancer, gastric cancer, ovarian cancer^325–327^	Promotes cell proliferation, migration, metastasis, EMT, metabolic flexibility and self-renewal of cancer stem cells, chemoresistance (miR-30a, miR-15b-5p)
Sirt2	/	promoter	Colorectal cancer lung cancer, renal cell carcinoma, gastric cancer, cervical cancer^328–330^	Highly expressed in stem-like cells and promotes migration, invasion and metastasis (p53, RAS/ERK/JNK/MMP-9 pathway)
		Suppressor	Breast cancer, prostate cancer lung cancer^331-333^	Sensitizes cancer cells to intracellular DNA damage and the cell death induced by oxidative stress, and low Sirt2 levels were associated with poor patient survival (p27)
Sirt3	/	Promoter	Cervical cancer, lung cancer^334,335^	Is associated with PD-L1-induced lymph node metastasis (p53)
		Suppressor	Pancreatic cancer, breast cancer, prostate cancer, gastric cancer, ovarian cancer^336–338^	Loss of SIRT3 leads to reactive oxygen species (ROS) generation that amplifies HIF-α stabilization; metastasis (*c-MYC*, CagA, PI3K/Akt pathway, Wnt/β-catenin pathway, AMP-activated protein kinase (AMPK))
Sirt4	/	Suppressor	Pancreatic cancer, thyroid cancer, gastric cancer, colorectal cancer^339,340^	Promotes cell proliferation, aerobic glycolysis, migration and invasion, and in inhibition of glutamine metabolism (E-cadherin)
Sirt5	/	Promoter	Colorectal cancer, lung cancer, breast cancer^341–343^	Promotes autophagy, cell proliferation, and drug resistance, and is associated with poor clinical outcomes
		Suppressor	Liver cancer, gastric cancer^344,345^	Inhibits peroxisome-induced oxidative stress (CDK2)
Sirt6	/	Promoter	Pancreatic cancer, lung cancer, prostate cancer^346–348^	Enhances cytokine production, and promotes EMT, cell migration and tumor metastasis, and predicts poor prognosis (ERK1/2/MMP9 pathway, SIRT6/Snail/KLF4 axis)
		Suppressor	Pancreatic cancer, breast cancer, liver cancer^349,350^	Promotes increased glycolysis, cancer cell proliferation and tumor growth, and is associated with paclitaxel, epirubicin, and trastuzumab sensitivity (survivin, NF-κB pathway)
Sirt7	/	Promoter	Colorectal cancer, gastric cancer, bladder cancer^351,352^	Accelerates cell growth, proliferation, motility and apoptosis (MAPK pathway)
		Suppressor	Pancreatic cancer, breast cancer, lung cancer, colorectal cancer^353–355^	Sensitizes to gemcitabine and radiotherapy, and low levels of SIRT7 are associated with an aggressive tumor phenotype and poor outcome (TGF-β signaling, p38 MAPK)
HDAC Class IV				
HDAC11	/	Promoter	Liver cancer, Hodgkin lymphoma, neuroblastoma, colorectal cancer, prostate cancer, breast cancer, ovarian cancer^356–359^	Promotes the mitotic cell cycle, cell apoptosis; is associated with cancer progression and survival (OX40 ligand, p53)

*EMT* epithelial-mesenchymal transition, *PI3K* phosphatidylinositol 3-kinase, *TGF-β* transforming growth factor β, *ER* estrogen receptor, *CSF* colony-stimulating factor, *AR* androgen receptor, *MMP* matrix metalloproteinase

#### Acetyl-lysine recognition proteins

The bromodomain (BRD) motif is an ~110-amino-acid conserved protein module and is regarded as the first and sole histone-binding module that contains a hydrophobic pocket to identify acetyl-lysine.^191^ The specificity of different BRDs depends on the sequences within the loops that form the hydrophobic pocket. Therefore, each BRD has a preference for different histones.^192,193^ In addition to their recognition of acetyl-lysine, BRDs are also capable of interacting with other chromatin molecules, such as plant homeodomain (PHD) finger motifs or another BRD. To date, 42 proteins containing bromodomains and 61 unique bromodomains have been discovered.^194,195^ Based on the sequence length and sequence identity of BRDs, the human BRD family can be divided into nine groups and one additional set of outliers, which has been well illustrated in published papers.^169,194^ Different BRD-containing proteins contain one to six BRDs. Intriguing, the most notable and well-studied bromodomain proteins are also HATs, such as PCAF, GCN5, and p300/CBP. Yaf9, ENL, AF9, Taf14, Sas5 (YEATS), and double PHD finger (DPF) have also been discovered to be acyl-lysine reader domains.^191,196^ Human MOZ and DPF2 are two proteins containing the DPF domain. Mutations in the YEATS and DPF domains are associated with cancer. For example, mutation of AF9 has been found in hematological malignancies, and ENL dysregulation leads to kidney cancer.^197,198^

Another important family is the BRD and extraterminal domain (BET) protein family, including BRD2, BRD3, BRD4, and BRDt, and this family shares two conserved N-terminal bromodomains and a more divergent C-terminal recruitment domain.^199,200^ These bromodomain proteins are critical as mediators of gene transcriptional activity.^201^ Of note, bromodomains have also been found in some histone lysine methyltransferases, such as ASH1L and MLL. BRDs are promiscuous domains and have been discussed in other well-constructed papers.^169,194^ In this review, we focus on the role of BRDs in tumorigenesis.

As histone acetylation “readers”, bromodomain proteins play important roles in tumorigenesis. BRD4 recruits the positive transcription elongation factor complex (P-TEFb), a validated target in chronic lymphocytic leukemia associated with *c-Myc* activity.^202–204^ Chromosomal translocation of BRD4, via the t(15;19) translocation, results in the generation of the fusion protein BRD4-NUT (nuclear protein in testis), which is found in NUT midline carcinoma (NMC). Importantly, inhibition of BRD4-NUT induces differentiation of NMC cells.^205^ Moreover, BRD4 is required for the maintenance of AML with sustained expression of Myc^206^ (Table 2).

#### Histone deacetylases

Histone deacetylases (HDACs) have recently attracted increasing attention. In humans, the genome encodes 18 HDACs. In contrast to the function of HATs, HDACs usually act as gene silencing mediators and repress transcription. Similarly, HDACs are expressed not only in the nucleus but also in the cytoplasm, and their substrates are also not limited to histones. Based on sequence similarity, HDACs can be divided into four classes: class I HDACs, yeast Rpd3-like proteins, are transcriptional corepressors and have a single deacetylase domain at the N-terminus and diversified C-terminal regions (HDAC1, HDAC2, HDAC3, and HDAC8); class II HDACs, yeast Hda1-like proteins, have a deacetylase domain at a C-terminal position (HDAC4, HDAC5, HDAC6, HDAC7, HDAC9, and HDAC10); class III HDACs are yeast silent information regulator 2 (Sir2)-like proteins (SIRT1, SIRT2, SIRT3, SIRT4, SIRT5, SIRT6, and SIRT7); and class IV involves one protein (HDAC11). The class IV protein shares sequence similarity with both class I and class II proteins.^207,208^ Classes I, II, and IV are included in the histone deacetylase family, whereas class III HDACs belong to the Sir2 regulator family.^209^ The catalytic mechanisms for these two families are different; classes I, II, and IV are Zn^2+^-dependent HDACs, whereas sir2-like proteins (sirtuins) are nicotinamide adenine dinucleotide (NAD^+^)-dependent HDACs and are also capable of mono-ADP-ribosyltransferase activity, another pattern of histone modification.^210^ Intriguingly, SIRT4 is thought to have more mono-ADP-ribosyltransferase activity than HDAC activity. SIR2 and SIRT6 seem to have equal levels of both mono-ADP-ribosyltransferase and HDAC activities.^211,212^ Moreover, after revealing the crystal structure of SIRT5, researchers found that SIRT5 is also a lysine desuccinylase and demalonylase.^213^ Therefore, the diversity of the sirtuin family makes them a group of multifunctional enzymes.

So far, the major known recognition sites of each HDAC are different, and these largely remain to be uncovered. For example, HDAC3 is thought to deacetylate H4K8 and H4K12,^214^ but in an HDAC3-knockout HeLa cell line, the acetylation levels of H4K8 and H4K12, even the overall acetylation levels of H3 and H4, were comparable with those in wild-type cells.^215^ Nevertheless, HDAC1 or HDAC3 siRNA can indeed increase the acetylation levels of H3K9 and H3K18.^215^ Therefore, partially because of the functional complementation and diversity within HDAC families, especially in class I, II, and IV, it is difficult to identify the specific substrates of certain HDACs. However, the substrates of the sirtuin family are quite clear. It is notable that because SIRT4 and SIRT5 are only located in mitochondria, they have no effect on histones. However, nonhistone lysine acetylation is also prevalent, since more than 3600 acetylation sites on 1750 proteins have been identified.^166^ The tumor suppressor p53 and the cytoskeletal protein α-tubulin are two representative substrates of HDACs.^216–218^ Notably, HDACs are also capable of regulating gene transcription by deacetylating other proteins that are responsible for epigenetic events, such as DNMTs, HATs, and HDACs.^166,219^ Another phenomenon is that some HDACs have to form a complex along with other components to function as transcriptional corepressors, which provides ideas and methods to design novel HDAC inhibitors. The Sin3, NuRD, and CoREST complexes are three complexes containing HDAC1 and HDAC2. Studies have found that purified HDAC1 or HDAC2 without associated components shows fairly weak deacetylation activity in vitro.^220^ HDAC3 interacts with the corepressors SMRT/NCoR to form the functional complexes, which significantly increases HDAC3 activity. NCoR also interacts with HDAC1, HDAC2 and the class II deacetylases HDAC4, HDAC5, and HDAC7, but usually not in the form of a complex.^221,222^ Deleted in breast cancer 1 (DBC1) and active regulator of SIRT1 (AROS) are two proteins that are able to bind to SIRT1, whereas their interactions present opposite functions. The DBC1/SIRT1 complex inhibits the deacetylation activity of SIRT1, whereas the combination of AROS and SIRT1 stimulates the activity of SIRT1.^223,224^

HDACs not only are able to deacetylate histones and nonhistone proteins but also interact with other epigenetic-associated enzymes, which gives them a vital role in tumorigenesis.^162,178^ Alterations in HDACs in cancers usually result in aberrant deacetylation and inactivation of tumor suppressor genes. For example, hypoacetylation of the promoter of p21, a tumor suppressor encoded by *CDKN1A*, can be reversed by HDAC inhibitors, resulting in an antitumor effect.^225^ A screen of the mutations in several HATs and HDACs, such as CBP, PCAF, HDAC1, HDAC2, HDAC5, HDAC7, and SIRT1, in more than 180 cancer samples including primary tumors and cancer cells indicated that the expression profiles of HDAC1, HDAC5, HDAC7, and SIRT1 are distinctive for colorectal cancers and normal colorectal mucosa, and the expression profiles of HDAC4 and CBP are capable of distinguishing breast cancer tissue from normal tissues^226^ (Table 2).

### Histone methylation (lysine and arginine)

Similar to the process of histone acetylation, histone methylation also consists of three important components: “writers”, histone methyltransferases (HMTs), “readers”, histone methylation-recognizing proteins, and “erasers”, histone demethylases (HDMs). Methylation of histones occurs at arginine and lysine residues. Arginine and lysine both can be monomethylated or dimethylated, whereas lysine is also capable of being trimethylated. Histone methylation can either promote or inhibit gene expression, which depends on the specific situation. For example, lysine methylation at H3K9, H3K27, and H4K20 is generally associated with suppression of gene expression, whereas methylation of H3K4, H3K36, and H3K79 induces gene expression.^360^ Mutation of H3K27M (lysine 27 to methionine) and H3K36M are two important oncogenic events, and H3K27M and H3K36M serve as drivers of pediatric gliomas and sarcomas. H3K27M has been identified in more than 70% of diffuse intrinsic pontine gliomas (DIPGs) and 20% of pediatric glioblastomas, which results in a global reduction in the trimethylation of H3K27 (H3K27me3).^361–363^ However, the H3K36M mutation impairs the differentiation of mesenchymal progenitor cells and generates undifferentiated sarcoma, leading to increased levels of H3K27me3 and global loss of H3K36 (me2 and me).^364,365^ Meanwhile, depletion of H3K36 methyltransferases results in similar phenotypes to those seen with H3K36M mutation.^364^ To date, KMTs (lysine methyltransferases) have been better studied than arginine methyltransferases (PRMTs) due to their sequence of discovery, different prevalence and impact. Their targets are not limited to only histones, they also modify other key proteins, such as the tumor suppressor p53, TAF10, and Piwi proteins.^366–368^

#### Histone methyltransferases

All KMTs contain a 130-amino-acid conserved domain, the SET (suppressor of variegation, enhancer of Zeste, trithorax) domain, except for DOT1L. The SET domain is responsible for the enzymatic activity of SET-containing KMTs. Instead of methylating lysine residues in histone tails, DOT1L methylates lysine in the globular core of the histone, and its catalytic domain is more similar to that of PRMTs.^369,370^ The enzymatic activity of KMTs results in the transfer of a methyl group from S-adenosylmethionine (SAM) to a the ε-amino group of a lysine residue. The first identified KMT was SUV39H1, which targets H3K9.^371^ Sequentially, more than 50 SET-containing proteins have been identified with proven or predicted lysine methylation potential. Of note, KMTs are highly specific enzymes, meaning that they are highly selective for lysine residues they can methylate and the specific methylation degree they can achieve. For example, SUV39H1 and SUV39H2 specifically methylate histone 3 at lysine 9 (H3K9), and DOT1L only methylates H3K79.^371^ Based on their structure and sequence around the SET domain, generally, KMTs can be divided into six groups, SUV39, SET1, SET2, EZH, SMYD, and RIZ (PRDM) (reviewed by Volkel and Angrand^372^). The Pre-SET domain of the SUV39 family contains nine conserved cysteines that coordinate with three zinc ions to function. The SET1 family members share a similar Post-SET motif that contains three conserved cysteine residues. The SET2 family possesses an AWS motif that contains 7–9 cysteines. Their SET domain is located between the AWS motif and a Post-SET motif. The members of the enhancer of zeste homolog (EZH) family are the catalytic components of polycomb repressive complexes (PRCs), which are responsible for gene silencing. EZH proteins have no Post-SET motif but have 15 cysteines in front of the SET domain and show no methylated activity as isolated proteins.^373^ PRC2 shows lysine methylation activity through its catalytic components, EZH2 or its homolog EZH1.^374^ EZH2 can methylate not only histone H3 but also histone H1 at lysine 26.^375^ The SMYD family members, which are SET and MYND domain-containing proteins, possesses a MYND (myeloid-nervy-DEAF1) domain, a zinc-finger motif responsible for protein–protein interaction.^376^ The RIZ (PRDM) family is a large family containing a homolog of the SET domain, the PR domain. The PR and SET domains share 20–30% sequence identity and are both capable of inducing histone H3 methylation.^377^ However, most members of the RIZ family responsible for histone methylation are still unknown. So far, two of them have been proven to induce the methylation of histones: PRDM2 (RIZ1) is associated with H3K9 methylation; and Meisetz, the mouse homolog of PRDM9, trimethylates H3K4.^378^ Meanwhile, PRDM1 has been identified to interact with EHMT2, a member of the SUV39 family. PRDM6 acts as a transcription suppressor by interacting with class I HDACs and EHMT2 to induce cell proliferation and inhibit cell differentiation.^379^ Meanwhile, the recruitment of EHMT2 is based on the formation of a complex with PRDM1.^380^ Due to the lack of a characteristic sequence or structure flanking the SET domain, other SET-containing KMTs, such as SET7/9, SET8, SUV4-20H1, and SUV4-20H2, cannot be classified into these families. Notably, some KMTs contain more than one domain, which allows them to interact with other proteins, especially other epigenetic modifying proteins. SUV39H1 possesses a chromodomain that directly binds to nucleic acids and forms heterochromatin.^381^ MLL1 recognizes unmethylated DNA through its CpG-interacting CXXC domain. SETDB1 contains an MBD that interacts with methylated DNA.^382^ The Tudor domain in SETDB1 may potentially recognize the methylation of lysine residues.^383^ ASH1 is able to interact with CBP, a HAT, via a bromodomain within ADH1.^384^

Protein arginine methyltransferases (PRMTs) can be divided into two groups. Among the nine PRMTs, only PRMT5, PRMT7, and PRMT9 are type II PRMTs, and the other five PRMTs, except for PRMT2, are type I PRMTs. PRMT2 was identified by sequence homology^385^ but has not shown any catalytic activity during investigations, although PRMT2 acts as a strong coactivator for androgen receptor (AR), which is thought to be associated with arginine methylation.^386^ Both types of PRMTs first catalyze the formation of monomethylarginine as an intermediate. However, sequentially, type I PRMTs can form asymmetric dimethylarginine (ADMA, Rme2a), but type II PRMTs form symmetric dimethylarginine (SDMA, Rme2s). Rme2a means two methyl groups on one ω-amino group, whereas an Rme2s has one methyl group on each ω-amino group. PRMT1-PRMT8 were investigated by Herrmann and Fackelmayer,^387^ and FBXO11 was identified as PRMT9, which symmetrically dimethylates arginine residues.^388^

Most enzymes for histone methylation are substrate-specific proteins; therefore, alterations in the aberrant expression of enzymes are usually associated with specific histone residue mutations. One of the best-known examples of alterations in tumorigenesis is H3K4me3, which is associated with biphenotypic (mixed lineage) leukemia (MLL). The location of the MLL gene is where chromosomal translocations in AML and ALL usually occur.^389^ When the MLL gene is translocated, the catalytic SET domain is lost, which results in MLL translocation-generated fusion proteins, which recruit DOT1L.^390^ Maintenance of MLL-associated ALL depends on the methylation of H3K79 catalyzed by DOT1L.^391^ Therefore, DOT1L is usually associated with hematological malignancies rather than solid tumors. Alteration of the EZH2-induced methylation of H3K27 has been observed in multiple cancers, including various solid tumors (prostate, breast, kidney, bladder, and lung cancers) and hematological malignancies.^392^ Meanwhile, overexpression of EZH2 has been found in multiple cancers and is associated with poor prognosis.^393^ Different mechanisms have been proposed to describe the role of EZH2 in tumorigenesis (Table 3).

**Table 3:** Important enzymes or proteins that regulate histone methylation in cancer.

Enzymes	Synonyms	Role in cancer	Cancer type	Mechanism
*Histone methyltransferases (lysine): the writers for lysine*				
SUV39				
KMT1A	SUV39H1, MG44, SUV39H	Promoter	Gastric cancer, prostate cancer, breast cancer, lung cancer, colorectal cancer, bladder cancer^421–426^	Promotes cell migration and cancer stem cell self-renewal (KMT1A-GATA3-STAT3 axis)
		Suppressor	Breast cancer, cervical cancer^427,428^	SUV39H1-low tumors are correlated with poor clinical outcomes
KMT1B	FLJ23414, SUV39H2	Promoter	Colorectal cancer, lung cancer, gastric cancer^429–431^	Promotes cell proliferation, migration and invasion and tumor metastasis
KMT1C	EHMT2, G9A, BAT8, NG36	Promoter	Breast cancer, pancreatic cancer, bladder cancer, ovarian cancer, liver cancer, colon cancer, lung cancer^432–435^	Promotes cell proliferation, metastasis, and apoptosis, and is associated with poor prognosis (p27, PMAIP1-USP9X-MCL1 axis, Wnt signaling pathway)
KMT1E	SETDB1, ESET, KG1T	Promoter	Breast cancer, colorectal cancer, hepatocellular carcinoma, liver cancer^436–439^	SETDB1 promotes cell proliferation, migration, invasion, and EMT (p53)
		Suppressor	Lung cancer^440^	SETDB1 acts as a metastasis suppressor, and inhibits cell migration and invasive behavior.
SET1				
KMT2A	MLL1, HRX, TRX1, ALL-1	Promoter	Head and neck cancer, pancreatic cancer, prostate cancer^441,442^	Promotes PD-L1 transcription and is associated with the self-renewal of cancer cells (Wnt/β-catenin pathway)
KMT2B	ALR, MLL2	promoter	Bladder cancer, lung cancer, breast cancer^443–445^	Is associated with the self-renewal of CSCs and expansion (Wnt/β-catenin pathway)
KMT2C	MLL3, HALR	Suppressor	Colorectal cancer, esophageal squamous cell carcinoma^446^	Inhibits tumor growth and metastasis
KMT2D	MLL4, HRX2	Promoter	Breast cancer^447^	Promotes cell proliferation and invasiveness
KMT2E	MLL5	Promoter	Glioblastoma^448^	Is associated with cancer cell self-renewal
KMT2F	SET1A	Promoter	Liver cancer^449^	Promotes liver cancer growth and hepatocyte-like stem cell malignant transformation
EZH				
EZH1	KIAA0388	Promoter	Breast cancer, prostate cancer, bladder cancer, colorectal cancer, liver cancer, gastric cancer, melanoma, lymphoma, myeloma, Ewing’s sarcoma, glioblastoma, thyroid carcinoma, esophageal squamous cell carcinoma, lung cancer, ovarian cancer, renal cancer^392,450–452^	Promotes cell proliferation, colony formation, migration and tumor metastasis; is associated with cancer stem cell maintenance; predicts chemotherapeutic efficacy and response to tamoxifen therapy (E-cadherin, RUNX3, MEK-ERK1/2-Elk-1 pathway)
EZH2	KMT6, ENX-1, MGC9169			
SET2				
KMT3A	SETD2, SET2, HIF-1,	Suppressor	Renal cancer, lung cancer^453,454^	Maintains genome integrity and attenuates cisplatin resistance (ERK signaling pathway)
WHSC1	NSD2, WHS, TRX5	Promoter	Prostate cancer, gastric cancer^455,456^	Promotes cell invasive properties, EMT and cancer metastasis
WHSC1L1	NSD3, MGC126766	Promoter	Breast cancer, head and neck cancer^457^	Is associated with ERα overexpression and enhances the oncogenic activity of EGFR
RIZ (PRDM)				
PRDM1	BLIMP1	Promoter	Pancreatic cancer, breast cancer^458,459^	Promotes cell invasiveness and cancer metastasis
		Suppressor	Lung cancer, colon cancer^460,461^	Inhibits cell invasion and metastasis (p21)
PRDM2	RIZ	Promoter	Colorectal cancer, breast cancer^462,463^	Is associated with poor clinicopathological variables and mediates the proliferative effect of estrogen
PRDM3	EVI1, MDS1-EVI1	Promoter	Ovarian cancer, nasopharyngeal carcinoma^464,465^	Promotes cell proliferation, migration, EMT, cancer stem cells and chemoresistance/radioresistance
PRDM4	PFM1	Promoter	Breast cancer^466^	Is associated with cancer cell stemness, tumorigenicity, and tumor metastasis
PRDM5	PFM2	Suppressor	Colorectal cancer, gastric cancer, cervical cancer^467^	Among the PRDM family genes tested, PRDM5 was the most frequently silenced in colorectal and gastric cancer
PRDM9	PFM6	Promoter	N/A^468^	Impairs genomic instability and drives tumorigenesis
PRDM14	PFM11	Promoter	Testicular cancer, pancreatic cancer^469,470^	Is associated with early germ cell specification and promotes cancer stem-like properties and liver metastasis
PRDM16	MEL1, PFM13	promoter	Gastric cancer^471^	Inhibits TGF-beta signaling by stabilizing the inactive Smad3-SKI complex
SMYD				
KMT3C	SMYD2	Promoter	Pancreatic cancer, gastric cancer, breast cancer, lung cancer^472,473^	Promotes cancer cell proliferation and survival (STAT3, EML4-ALK, p65)
KMT3E	SMYD3, ZMYND1, ZNFN3A1, FLJ21080	Promoter	Liver and colon cancer, prostate cancer, breast cancer^474–476^	Promotes cell proliferation, invasion, EMT and cancer stem cell maintenance (Myc, MMP-9, Ctnnb1, JAK/Stat3 pathway, Wnt pathway, androgen receptor transcription)
SMYD4	ZMYND21	Suppressor	Breast cancer^477^	SMYD4 acts as a suppressor in tumorigenesis
Others				
DOT1L	KMT4	promoter	MLL-rearranged leukemia, colorectal cancer, breast cancer, ovarian cancer^391,478,479^	Increases EMT, cancer stemness and tumorigenic potential and is required for MLL rearrangement
SET8	KMT5A, SETD8, PR-set7	promoter	Breast cancer, prostate cancer, ovarian cancer, lung cancer^480,481^	Promotes cell proliferation, migration, invasion, and EMT (MiR-502)
SUV4-20H2	KMT5C, MGC2705	Suppressor	Breast cancer^482^	SUV4-20H2 is downregulated in breast cancer
SetD6	/	Promoter	Colorectal cancer, bladder cancer, breast cancer^483,484^	Promotes cell survival and colony formation and contributes to increased susceptibility to cancer
SET7/9	SETD7, KMT7	Suppressor	Breast cancer, gastric cancer, AML, lung cancer^485–487^	Promotes cell proliferation, EMT and the generation of cancer stem cells; a low level of SET7/9 is correlated with clinical aggressiveness and worse prognosis (β-catenin stability)
*Histone methyltransferases (arginine): the writers for arginine*				
PRMT1	ANM1, HCP1, IR1B4	Promoter	Breast cancer, colon cancer, gastric cancer, lung cancer^488–490^	Promotes EMT, cancer cell migration, and invasion and is associated with chemosensitivity and poor clinical and histological parameters
		Suppressor	Pancreatic cancer^491^	Inhibits cell proliferation and invasion in pancreatic cancer
PRMT2	/	Suppressor	Breast cancer^492^	Induces cell cycle arrest and apoptosis in breast cancer
PRMT4	CARM1	Promoter	Ovarian cancer, breast cancer, liver cancer, colorectal cancer, prostate cancer^450,493,494^	Promotes cell proliferation and blocks cell differentiation (Wnt/β-catenin signaling)
		Suppressor	Pancreatic cancer^495^	Inhibits glutamine metabolism and suppresses cancer progression
PRMT5	JBP1, SKB1, IBP72	Promoter	Breast cancer, prostate cancer, colorectal cancer, lung cancer^496–498^	Promotes cell survival, proliferation, invasiveness and sensitivity to 5-Fluorouracil (5-FU) (SHARPIN-PRMT5-H3R2me1 axis)
		Suppressor	Breast cancer^499^	High PRMT5 expression favors a better prognosis in BC patients
PRMT6	HRMT1L6	Promoter	Prostate cancer, gastric cancer^500,501^	Is associated with cell apoptosis, invasiveness and viability (PI3K/AKT/mTOR pathway, H3R2me2as)
		Suppressor	Hepatocellular carcinoma^502^	Negatively correlates with aggressive cancer features
PRMT7	FLJ10640, KIAA1933	Promoter	Lung cancer, breast cancer^503,504^	Promotes cancer cell EMT and tumor metastasis
PRMT8	HRMT1L3, HRMT1L4	Promoter	Breast, ovarian and gastric cancer^505^	Overexpression of PRMT8 is correlated with decreased patient survival
PRMT9	FBXO11	Promoter	Breast cancer^506^	Fuels tumor formation via restraint of the p53/p21 pathway
*Methyl-histone recognition proteins: the readers*				
Chromodomain				
HP1	/	Promoter	Breast cancer^507^	Overexpression of HP1 is associated with breast cancer progression
Chd1	/	Promoter	Prostate cancer^508^	Is associated with cell invasiveness, double-strand break repair and response to DNA-damaging therapy
		Suppressor	Prostate cancer^509^	Loss of MAP3K7 and CHD1 promotes an aggressive phenotype in prostate cancer
WD40 repeat domain				
WDR5	/	/	/	
MBT domain				
BPTF	/	Promoter	Lung cancer, hepatocellular carcinoma^510,511^	Promotes cell proliferation, migration, stem cell-like traits and invasion (miR-3666)
L3MBTL1	/	Suppressor	Breast cancer^512^	Expression of L3MBTL1 is associated with a low risk of disease recurrence and breast cancer-related death
ING2		Promoter	Colon cancer^513^	Increases invasion by enhancing MMP13 expression
		Suppressor	Lung cancer^514^	Suppresses tumor progression via regulation of p53
BHC80		Promoter	Prostate cancer^515^	Stimulates cell proliferation and tumor progression via the MyD88-p38-TTP pathway
*Tudor domains*				
JMJD2A		Promoter	Breast cancer, liver cancer, colon cancer^516,517^	Promotes cells apoptosis and proliferation and contributes to tumor progression (ARHI, miR372)
		Suppressor	Bladder cancer^518^	Low JMJD2A correlates with poor prognostic features and predicts significantly decreased overall survival
*KDMs: the erasers*				
KDM1				
KDM1A	LSD1	Promoter	Breast cancer, lung cancer, prostate cancer, liver cancer, pancreatic cancer, gastric cancer^519–521^	Contributes to cell proliferation and stem cell maintenance and self-renewal (p21, AR, HIF1α-dependent glycolytic process)
		Suppressor	Breast cancer^522^	Inhibits invasion and metastatic potential
KDM1B	LSD2	Promoter	Breast cancer^523^	Contributes to cancer progression and cancer stem cell enrichment
KDM2/JHDM1				
KDM2A	JHDM1A, CXXC8	Promoter	Breast cancer, gastric cancer, lung cancer, cervical cancer^524–526^	Promotes cancer cell proliferation, metastasis, and invasiveness (HDAC3, TET2)
KDM2B	JHDM1B, FBXL10,	Promoter	Prostate cancer, breast cancer, gastric cancer^527,528^	Promotes cell migration, angiogenesis, and the self-renewal of cancer stem cells
KDM3/JHDM2/JMJD1				
KDM3A	JHDM2A, JMJD1A	Promoter	Colorectal cancer, ovarian cancer, breast cancer, prostate cancer, bladder cancer^529–531^	Promotes cancer cell growth, metastasis, stemness and chemoresistance (*c-Myc*, Wnt/β-catenin signaling, glycolysis, HIF1α)
KDM3C	JHDM2C, JMJD1C	Promoter	Esophageal cancer, colorectal cancer^532,533^	Promotes cancer cell proliferation and metastasis (YAP1 signaling, ATF-2)
KDM4/JHMD3/JMJD2				
KDM4A	JHDM3A, JMJD2A	Promoter	Breast cancer, liver cancer^516,534^	Promotes cancer progression through repression of the tumor suppressor ARHI (miR372)
		Suppressor	Bladder cancer^518^	Downregulated in cancer tissues and significantly decreases as cancer progresses
KDM4B	JMJD2B	Promoter	Breast cancer, gastric cancer, ovarian cancer, colorectal cancer, prostate cancer^535–537^	Promotes EMT and metastasis, and regulates the seeding and growth of peritoneal tumors; is involved in resistance to PI3K inhibition (p-ERK, β-catenin)
KDM4C	JMJD2C, GASC1	Promoter	Breast cancer, pancreatic cancer^538,539^	Promotes cancer progression (HIF-1α, miR-335-5p)
KMD4D	JMJD2D	Promoter	Colorectal cancer^540^	Promotes cell proliferation and tumor growth (β-catenin)
KDM5/JARID				
KDM5A	JARID1A, RBP2	Promoter	Breast cancer, colorectal cancer, cervical cancer^541,542^	Promotes proliferative activity and invasion, and inhibition of KDM5A causes growth arrest at the G1 phase (*c-Myc*)
KDM5B	JARID1B, RBP2-like	Promoter	Colorectal cancer, lung cancer, gastric cancer^543^	Promotes cell proliferation, metastasis, and expression of CSCs, and inhibition of KDM5B results in cell cycle arrest, apoptosis, and senescence (E2F/RB pathway)
KDM5C	JARID1C, SMCX	Promoter	Prostate cancer, lung cancer^544^	Overexpression of KDM5C predicts therapy failure and is associated with cancer cell growth, migration and invasion
		Suppressor	Colon cancer^545^	Inhibits the multidrug resistance of colon cancer cell lines by downregulating ABCC1
KDM5D	JARID1D, SMCY	Promoter	Gastric cancer^546^	Promotes cell proliferation and EMT
		Suppressor	Prostate cancer^547^	Loss of KDM5D expression induces resistance to docetaxel
JARID2	JUMONJI	Promoter	Bladder cancer, lung and colon cancers^548,549^	Regulates cancer cell EMT and stem cell maintenance and is associated with poor survival
		Suppressor	Prostate cancer^550^	Inhibits cell proliferation, migration, and tumor development via inhibition of Axl
KDM6/UT				
KDM6A	UTX	Promoter	Breast cancer^447^	Promotes cell proliferation and invasiveness
		Suppressor	Bladder cancer, pancreatic cancer^551,552^	KDM6A loss induces squamous-like, metastatic pancreatic cancer
KDM6B	JMJD3	Promoter	Ovarian cancer, breast cancer, gastric cancer^553,554^	High expression of KDM6B is correlated with poor prognosis
KDM6C	UTY	Suppressor	Bladder cancer^555^	UTY-knockout cells have increased cell proliferation compared to wild-type cells
KDM7/PHF				
KDM7A	JHDM1D	Promoter	Prostate cancer^556^	Promotes cell proliferation and upregulated androgen receptor activity
KDM7C	PHF2, JHDM1E	Suppressor	N/A^420^	Is a suppressor and promotes p53-driven gene expression
KDM7B	PHF8, JHDM1F	Promoter	Prostate cancer, gastric cancer, lung cancer, leukemia, colorectal cancer^557–559^	Promotes cell proliferation, migration and invasion, and high PHF8 expression predicts poor survival (miR-488)
Others				
JMJD5	KDM8	Promoter	Breast cancer^560,561^	Promotes metastasis and indicates a poor prognosis; is required for cell cycle progression via because of its actions in the cyclin A1 coding region.
RSBN1	KDM9	Promoter	Breast cancer^562^	Is a new potential HIF target
JMJD6	PSR, PTDSR	Promoter	Breast cancer, oral cancer, lung cancer^563–565^	Promotes cancer cell proliferation, EMT and motility, and maintains cancer cell stemness properties (autophagy pathway, WNT/β-catenin pathway)
PADI4	/	Promoter	Breast cancer, esophageal cancer^566^	Promotes cancer progression and is correlated with pathological classification (c-Fos)

*EMT* epithelial-mesenchymal transition, *CSC* cancer stem cell, *EGFR* epidermal growth factor receptor, *MMP* matrix metalloproteinase, *PI3K* phosphatidylinositol 3-kinase

#### Methyl-histone recognition proteins

“Readers” of histone methylation contain several specific domains recognizing lysine or arginine methylation, such as a chromodomain,^394^ the WD40 repeat, the MBT (malignant brain tumor) domain, the Tudor domain^395^ and the PHD (plant homeodomain) finger motif.^396^ Representative chromodomain-containing proteins in humans are HP1 and Chd1, which can recognize H3K9me and H3K27me, respectively.^394,397^ WDR5 is a protein containing WD40 repeats. In addition to H3K4me, WDR5 prefers to bind to H3K4me2 via a histone-methylating complex and is required for maintaining H3K4me3.^395^ Later, WDR5 was shown to directly read H3R2, a “WIN” motif of MLL1, as well as symmetrical H3R2 dimethylation through the WD40 domain.^398^ L3MBTLs are a group of proteins containing three MBT repeat domains. L3MBTL1 represses gene expression via monomethylation or dimethylation of H4K20 or H1BK26.^399^ BPTF, RAG2, PYGO, and the tumor suppressor ING2 are representative proteins containing PHD finger motifs. They are all able to recognize and bind to H3K4me3.^400^ Intriguingly, DNMT3L and BHC80 also possess a PHD finger motif, but they selectively bind to unmethylated H3K4.^401,402^ There are a number of proteins containing Tudor domains, with a representative protein being JMJD2A. JMJD2A is a histone demethylase that equally binds to H3K4me3 and H4K20me3^403^ (Table 3).

#### Histone demethylases

The identification of histone demethylases (HDMs or KDMs) has lagged behind that of HMTs. Thus far, KDMs can be classified into two groups. The amine-oxidase type lysine-specific demethylases (LSDs) and the highly conserved JumonjiC (JMJC) domain-containing histone demethylases. LSD1 and LSD2, also known as KDM1A and B, are flavin adenine dinucleotide (FAD)-dependent amine oxidases that can only demethylate monomethylated and dimethylated lysine residues. LSD1 has been identified to specifically activate androgen receptor (AR) target genes along with AR by demethylating H3K9.^404^ The human genome codes more than 30 JMJC-containing KDMs that are able to remove methyl groups from all three methyl-lysine states. JHDM1A was the first characterized JMJC domain-containing HDM and specifically demethylates H3K36me2 and H3K36me1.^405^ Not all JMJC domain-containing proteins are able to demethylate histone proteins, such as HIF1AN and the transmembrane phosphatidylserine receptor PTDSR. JMJC-containing HDMs can be divided into six families:^360^ the JHDM1, JHDM2 (JMJD1), JHMD3 (JMJD2), JARID, PHF, and UT families. Notably, not all of these families possess the ability of histone demethylation. However, some JMJC-containing proteins, including those that are not included in these six families, contain one or more methylated-histone-binding domains. Their potential to demethylate methyl-lysine or methyl-arginine must be investigated. In addition to demethylases for lysine residues, JMJD6 is the first described arginine demethylase and lysine hydroxylase. It can remove methyl groups from H3R2 and H4R3.^406^ Another kind of protein is peptidylarginine deiminases (PADs or PADIs) or protein-arginine deiminases, which are able to convert arginine and monomethylated arginine to citrulline.^407^

LSD1 (KDM1A) is one of the best-studied KDMs and has been found to be increased in multiple cancers. Inhibition of LSD1 leads to global H3K4 methylation and promotes differentiation of neuroblastoma cells.^408^ Unlike KDM1A, KDM1B is mostly involved in growing oocytes with a restricted expression pattern.^409^ Similar to the dual roles of LSD1, members of the KDM2 family can either promote tumor formation or inhibit tumorigenesis.^410^ Through dimethylating H3K36 in DUSP3 (dual specific phosphatase 3), KDM2A activates ERK1/2 expression in lung cancer cells.^411^ Knockout of KDM2B in breast cancer downregulates the tumor stem cell markers ALDH and CD44 via the repression of polycomb complexes. KDM2B is also overexpressed in pancreatic ductal adenocarcinoma (PDAC) and cooperates with KrasG12D to promote PDAC formation in mouse models.^412^ The LSD1 and KDM2 family possesses context-dependent tumor-promoting and -inhibiting functions, which might depend on the different features of various cancers and the specific substrates of the enzymes. Therefore, further studies should take the dual roles of these enzymes into consideration. KDM3A, induced by hypoxia and nutrient starvation within the tumor microenvironment, shows carcinogenic effects via the promotion of tumor cell migration and invasion. Inhibition of KDM3A downregulates tumor-associated angiogenesis and macrophage infiltration.^413,414^ KDM3C is required for MLL-AF9 leukemia maintenance and is mutated in patients with intracranial germline tumors.^415,416^ KDM4A, KDM4B, and KDM4C have shown increased expression in prostate cancer with decreased levels of H3K9me2/3 and increased levels of H3K9me1.^417^ H3K9me3 is thought to be a hallmark of heterochromatic areas of the genome. In addition, KDM4 family members were the first identified demethylases targeting trimethylated lysines. Aberrant expression of KDM4 family members might lead to instability of the genome and become involved in tumorigenesis.^410^ Members of the KDM6 family usually act as tumor suppressors and are thought to cause cell growth arrest.^418^ For example, the tumor suppressor proteins p16INK4A and p14ARF, encoded by the INK4A-ARF locus, are repressed by H3K27me3. When stimulated by oncogenic factors, KDM6B is recruited to the INK4A-ARF locus and activates the transcription of these two tumor suppressors.^419^ In colorectal cancer, KDM7C is required for the efficacy of oxaliplatin and doxorubicin and for the activation of p53^420^ (Table 3).

## Noncoding RNA

Epigenetic related noncoding RNAs (ncRNAs) include microRNAs (miRNAs), small interfering RNA (siRNAs), Piwi-interacting RNA (piRNAs), and long noncoding RNAs (lncRNAs). MiRNAs, one of the most studied ncRNAs, are small RNAs between 19 and 22 nucleotides in length that play important roles in the regulation of gene expression by controlling mRNA translation. Intriguingly, the regions that miRNAs usually target are frequently associated with carcinogenesis.^567^ Generally, they can be divided into tumor-promoting and tumor-suppressing miRNAs. During tumorigenesis, oncogenic miRNAs such as miR-155, miR-21 and miR-17-92 are usually overexpressed, and tumor-suppressive miRNAs such as miR-15-16 are downregulated.^568^ There is another type of miRNA, cellular context-dependent miRNAs, functioning in tumorigenesis. For example, miR-146 has been shown to be overexpressed in multiple cancers, whereas a recent study has proven that miR-146 can reduce the expression of BRCA1.^568,569^ Meanwhile, the expression of proteins and enzymes is also regulated by certain miRNAs. MiR-101 directly represses EZH2, and abnormal downregulation of miR-101 has been observed in cancers.^570,571^ The expression of the miR-29 family is inversely correlated with that of DNMT3A and -3B in lung cancer tissues. Forced expression of miR-29 inhibits tumorigenesis by inducing reexpression of methylation-silenced tumor suppressor genes.^572^ LncRNAs are another large group of noncoding RNAs that play a vital role in tumorigenesis. Some lncRNAs are cancer type-specific, such as PCGEM1 in prostate cancer and HEIH in hepatocellular carcinoma.^573,574^ Many aberrant lncRNAs have been discovered in various cancers. Dysregulation of HOTAIR has been found in lung, pancreatic, and colorectal cancer.^575–577^

Therefore, ncRNAs can either be directly involved in tumorigenesis or indirectly affect tumor development by participating in other epigenetic events.

## Inhibitors and clinical trials

Unlike genetic mutations, epigenetic alterations are reversible. Given the importance of epigenetic marks in tumorigenesis, the availability of corresponding inhibitors has attracted extensive attention. Meanwhile, epigenetic regulation of a gene usually requires more than one epigenetic event. Currently, there are six epigenetic drugs approved for clinical use by the FDA (Table 4).

**Table 4:** Epigenetic drugs approved by the FDA.

Compound	Synonym	Clinical name	Condition	Approved year	Company
Azacitidine	5-Azacitidine, 5-Aza-CR	Vidaza	MDS	U.S. FDA (2004)	Pharmion Corporation
5-Aza-2′-deoxycytidine	5-Aza-CdR, decitabine	Dacogen	MDS	U.S. FDA (2006)	Janssen Pharmaceuticals
Suberoylanilide hydroxamic acid (SAHA)	Vorinostat	Zolinza	CTCL	U.S. FDA (2006)	Merck
Romidepsin	Depsipeptide, FK-229, FR901228	Istodax	CTCL	U.S. FDA (2009)	Celgene
Belinostat	PXD101	Beleodaq	PTCL	U.S. FDA (2014)	TopoTarget
Panobinostat	LBH589	Farydak	Multiple myeloma	U.S. FDA (2015)	Novartis
Chidamide	Tucidinostat, HBI-8000	Epidaza	PTCL	China FDA (2015)	Chipscreen Biosciences

*FDA* Food and Drug Administration, *MDS* myelodysplastic syndrome, *CTCL* cutaneous T-cell lymphoma, *PTCL* peripheral T-cell lymphoma

### Targeting DNA methylation

Blockade of DNMTs is the most effective way to prevent aberrant DNA hypermethylation. However, until now, targeting of the methyltransferase enzymes still lacks specificity and even causes hypomethylation of the global genome.^578^ Complete deletion of DNMT1 in mice results in embryonic lethality.^579^ Knockout of DNMT1 in fibroblast cells causes aberrant expression of 10% of genes and p53-dependent death.^580^ Administration of DNA methylation inhibitors results in tumorigenesis in male Fischer rats.^581^ Regulation of DNA methylation is vital in cell survival and function, and in addition to the specificity needed and the side effect associated, it is hard to identify proper drugs.

DNA methylation inhibitors can be divided into two groups: nucleoside analogs and nonnucleoside analogs. Nucleoside analogs have a modified cytosine ring and can be turned into nucleotides and incorporated into newly synthesized DNA or RNA. DNA methyltransferases are bound by covalent complexes with the analogs, which inhibits DNA methylation. 5-Azacitidine (5-Aza-CR) and 5-aza-2′-deoxycytidine (5-Aza-CdR) are currently the two most studied and promising demethylation agents.^582^ 5-Aza-CR and zebularine are ribonucleoside analogs that can be phosphorylated to be able to incorporate into RNA. However, they can also be incorporated into DNA via the ribonucleotide reductase pathway. 5-Azacitidine, an analog of cytidine, is an injectable suspension for the treatment of myelodysplastic syndromes (MDSs). It promotes cell differentiation, demethylation, and reexpression of inactivated genes.^583^ The 5-azacitidine side effects include fetal abnormalities^584^ and decreased male fertility, especially at high doses, but its analog, 6-azacytidine, does not show such effects.^585^ Notably, after treating the noninvasive breast cancer cell lines MCF-7 and ZR-75-1 with azacytidine, the cells gained invasive abilities due to the hypomethylation of several prometastasis genes.^586^ Decitabine (5-Aza-CdR) and 5-fluoro-2′-deoxycytidine (5-F-CdR) are deoxyribonucleoside analogs that are capable of incorporating into DNA following phosphorylation. Decitabine (5-aza-2′-deoxycytidine) inhibits DNA methylation in a dosage-dependent manner. It can reactivate silenced genes at low doses but gains cytotoxicity at high doses, while myelosuppression is the major side effect at all doses.^587^. Dihydro-5-azacytidine (DHAC) is a biologically active and chemically stable analog of 5-azacitidine with decreased toxicity.^588,589^ Because of its hydrolytic stability, it may be administrated via prolonged i.v. infusion, potentially eliminating the acute toxicities caused by administration of 5-azacytidine.^590^ Zebularine is a potential oral DNA-demethylating drug with stability in acidic environments and in aqueous solutions.^591^ However, the near millimolar dose requirements and the limited bioavailability in rodents (<7%) and primates (<1%) leave zebularine far from clinical translation.^592^

Among the drugs discussed, 5-Aza-CR^593^ and 5-Aza-CdR^594^ have already been approved by the US Food and Drug Administration (FDA) for the treatment of certain subtypes of MDS and chronic myelomonocytic leukemia. Because of their intrinsic preference for newly synthetic DNA, they tend to affect dividing cells, i.e., cancer cells.^595^ Ongoing preclinical experiments and clinical trials are exploring their efficacy in solid tumors. The common side effects of these nucleoside-like analogs are mutagenic risk and genomic instability. Nonnucleoside analogs are capable of avoiding these side effects.

Currently, many nonnucleoside analogs have been developed to prevent DNA from aberrant hypermethylation. These drugs are usually small molecular inhibitors and directly target catalytic sites rather than incorporating into DNA. Based on a three-dimensional model of DNMT1, RG108 was designed to block the activity of this enzyme and cause demethylation.^596^ Psammaplin is a group of natural extracts from the sponge *Pseudoceratina purpurea* and is capable of inhibiting both DNA methyltransferases and histone deacetylases with mild cytotoxicity.^597^ Similarly, EGCG ((-)-epigallocatechin-3-gallate) is the major polyphenol from green tea and reversibly demethylates methyl-DNA, resulting in the reactivation of multiple key genes, including hMLH1, P16, and RA, in colon, esophageal, and prostate cancer cell lines.^598^ Both hydralazine and procainamide, two drugs associated with lupus-like autoimmune diseases, can inhibit DNA methylation and induce self-reactivity in cloned T-cell lines.^599^ They have promising tumor suppressor-reactivating and antitumor actions in breast cancer.^600,601^ Another strategy is developing antisense oligonucleotides to inhibit DNMT transcription. MG98 is a second-generation phosphorothioate antisense oligodeoxynucleotide that prevents DNMT1 mRNA translation effects but has no obvious antitumor effect.^602^ It has been under investigation in preclinical experiments and phase I/II clinical trials, especially in solid tumors.^603,604^ Of note, in a systemic analysis comparing nonnucleoside inhibitors with 5-Aza-CdR, the latter showed better efficacy in DNA demethylation inhibition.^605^

To date, hundreds of clinical trials have investigated the effects of anti-DNA methylation therapy for various cancers (Table 5).

**Table 5:** Important ongoing clinical trials with DNA methylation-targeted therapies.

Condition	Design	Sample size	Phase	Current status	NCT
*Azacitidine (5-azacitidine)-based trials*					
High-risk MDS	Azacitidine	44	IV	Completed	NCT01201811
Low-risk MDS	Azacitidine	216	III	Active, not recruiting	NCT01566695
High-risk MDS	Azacitidine	358	III	Completed	NCT00071799
CML	Azacitidine	11	II	Completed	NCT01350947
AML, MDS	Azacitidine	187	III	Completed	NCT00887068
Relapsed or refractory T-cell lymphoma	Azacitidine	20	III	Recruiting	NCT03703375
AML with complete remission	Azacitidine	472	III	Active, not recruiting	NCT01757535
Recurrent IDH1/2-mutated glioma	Azacitidine	63	II	Not yet	
recruiting	NCT03666559				
Prostate cancer	Azacytidine	36	II	Completed	NCT00384839
Head and neck squamous cell carcinoma	Azacitidine	25	II	Recruiting	NCT02178072
Locally advanced or metastatic nasopharyngeal carcinoma	Azacitidine	36	II	Completed	NCT02269943
Pancreatic cancer	Azacitidine	80	II	Recruiting	NCT01845805
Solid tumors and hematological disorders	Azacitidine	125	II	Recruiting	NCT02494258
AML	Azacitidine + venetoclax	42	II	Recruiting	NCT03466294
AML	Azacitidine + venetoclax	30	II	Recruiting	NCT03573024
AML	Azacitidine + venetoclax	400	III	Recruiting	NCT02993523
AML, MDS	Azacitidine + eltrombopag	25	II	Completed	NCT01488565
MDS	Azacitidine + eltrombopag	356	III	Terminated	NCT02158936
MDS	Azacitidine + APR-246	156	III	Recruiting	NCT03745716
AML, MDS	Azacitidine + DLI	30	II	Completed	NCT01541280
AML/MDS	Azacitidine + lenalidomide	72	II	N/A	NCT01556477
High-risk MDS with 5q deletion	Azacytidine + lenalidomide	50	II	Completed	NCT01088373
AML	Azacitidine + lenalidomide	88	II	Completed	NCT01358734
Elderly patients with AML	Azacitidine + lenalidomide	120	II	Completed	NCT01301820
Refractory AML	Azacitidine + lenalidomide	37	II	Completed	NCT01743859
MDS, CMML and AML relapsing after allo-HSCT	Azacitidine + lenalidomide + DLI	50	II	Active, not recruiting	NCT02472691
MDS with excess blasts 2	Azacitidine + vosaroxin	168	II	Recruiting	NCT03338348
AML	Azacitidine vs conventional care regimen	488	III	Completed	NCT01074047
AML, MDS with FLT3-ITD mutation	Azacitidine + sorafenib	17	II	Completed	NCT02196857
Advanced solid tumors	Azacitidine + durvalumab	60	II	Recruiting	NCT02811497
High-risk MDS, AML	Azacitidine + durvalumab	213	II	Active, not recruiting	NCT02775903
MDS patients with excess blasts, progressing	Azacitidine + rigosertib	67	III	Active, not recruiting	NCT01928537
AML, MDS, CML	Azacitidine + HAG regimen	120	III	Not yet	
recruiting	NCT03873311				
Refractory or relapsed AML	Azacitidine + lirilumab	37	II	Completed	NCT02399917
AML	Azacitidine + induction therapy	336	II	N/A	NCT01180322
AML with NPM1 mutation	Azacitidine + pembrolizumab	28	II	Not yet recruiting	NCT03769532
Pancreatic cancer	Azacitidine + pembrolizumab	31	II	Recruiting	NCT03264404
Metastatic melanoma	Azacitidine + pembrolizumab	71	II	Recruiting	NCT02816021
MDS	Azacitidine + pembrolizumab	40	II	Recruiting	NCT03094637
Chemorefractory metastatic colorectal cancer	Azacitidine + pembrolizumab	31	II	Active, not recruiting	NCT02260440
Advanced or metastatic non-small-cell lung cancer	Azacitidine + pembrolizumab	100	II	Active, not recruiting	NCT02546986
Platinum-resistant ovarian cancer	Azacitidine + pembrolizumab	20	II	Recruiting	NCT02900560
MDS	Azacitidine + lintuzumab	7	II	Terminated	NCT00997243
Prostate cancer	Azacitidine + ATRA	20	II	Recruiting	NCT03572387
Recurrent or refractory disease with IDH2 mutation	Azacitidine + enasidenib	50	II	Recruiting	NCT03683433
High-risk MDS with IDH2 mutation	Azacitidine + enasidenib	105	II	Recruiting	NCT03383575
Elderly patients with AML	Azacitidine + standard therapy	214	II	Completed	NCT00915252
Refractory or relapsed AML	Azacitidine + avelumab	52	I/II	Recruiting	NCT02953561
AML, MDS, CML	Azacitidine + pevonedistat	450	III	Recruiting	NCT03268954
Relapsed or refractory AML	Azacitidine + pevonedistat	72	II	Not yet recruiting	NCT03745352
High-risk MDS, AML, CML	Azacitidine + pevonedistat	120	II	Active, not recruiting	NCT02610777
AML without remission after allogeneic stem cell transplantation	Azacitidine + pevonedistat	30	II	Recruiting	NCT03709576
MDS	Azacitidine + pevonedistat	71	II	Recruiting	NCT03238248
Elderly patients with AML	Azacitidine + gemtuzumab ozogamicin	133	II	Active, not recruiting	NCT00658814
Recurrent and resectable osteosarcoma	Azacitidine + nivolumab	51	I/II	Not yet recruiting	NCT03628209
Childhood relapsed/refractory AML	Azacitidine + nivolumab	26	I/II	Not yet recruiting	NCT03825367
Elderly patients with AML or high-risk MDS	Azacitidine/decitabine + nivolumab or midostaurin	1670	II/III	Suspended	NCT03092674
Refractory/relapsed AML	Azacitidine + ipilimumab + nivolumab	182	II	Recruiting	NCT02397720
MDS	Azacitidine + nivolumab + ipilimumab	120	II	Recruiting	NCT02530463
MDS	Azacitidine + lirilumab + nivolumab	12	II	Completed	NCT02599649
High-risk MDS, AML	Azacitidine + sirolimus	74	II	Recruiting	NCT01869114
AML with IDH1 mutation	Azacitidine + AG-120	392	III	Recruiting	NCT03173248
Relapsed/refractory diffuse large B-cell lymphoma	Azacitidine + rituximab	27	II	Not yet recruiting	NCT03719989
Leukemia	Azacitidine + PKC412	54	I/II	Completed	NCT01202877
MDS	Azacitidine + sonidegib	78	I	Active, not recruiting	NCT02129101
MDS, AML and CMML	Azacitidine + PF-04449913	102	II	Recruiting	NCT02367456
MDS	Azacitidine + etanercept	32	I/II	Completed	NCT00118287
MDS, myeloproliferative neoplasm	Azacitidine + ruxolitinib Phosphate	123	II	Completed	NCT01787487
Relapsed or refractory AML, MDS	Azacitidine + quizartinib	72	I/II	Recruiting	NCT01892371
AML	Azacitidine vs fludarabine + cytarabine	289	III	Active, not recruiting	NCT02319135
AML, high-risk MDS	Azacitidine + cytarabine + tosedostat	96	I/II	Active, not recruiting	NCT01636609
Peripheral T-cell lymphoma	Azacitidine + CHOP	20	II	Recruiting	NCT03542266
AML	Azacitidine + intensive chemotherapy	720	III	Recruiting	NCT03416179
Advanced non-small-cell lung cancer	Azacitidine + paclitaxel	240	II	Active, not recruiting	NCT02250326
*Decitabine (5-aza-2*′*deoxycytidine)-based trials*					
Refractory CML	Decitabine	40	II	Completed	NCT00042003
Metastatic papillary thyroid cancer or follicular thyroid cancer	Decitabine	12	II	Completed	NCT00085293
AML with TP53 mutation	Decitabine	60	II	Recruiting	NCT03063203
AML	Decitabine	546	II	Completed	NCT00416598
MDS	Decitabine	128	II	Completed	NCT00067808
Elderly patients with AML	Decitabine	238	II	Completed	NCT00866073
Advanced-stage MDS	Decitabine	160	III	Completed	NCT00043381
Relapse and refractory diffuse large B-cell lymphoma	Decitabine	60	IV	Recruiting	NCT03579082
Relapsed or refractory T lymphoblastic lymphoma	Decitabine	40	IV	Recruiting	NCT03558412
CML	Decitabine + imatinib mesylate	80	II	Completed	NCT00054431
High-risk MDS, AML	Decitabine + tosedostat	34	II	Completed	NCT01567059
Metastatic castration-resistant prostate cancer	Decitabine + enzalutamide	21	I/II	Not yet recruiting	NCT03709550
Peripheral T-cell lymphoma	Decitabine + CHOP	100	III	Not yet recruiting	NCT03553537
Relapsed FLT3-ITD-mutated AML, MDS	Decitabine + quizartinib	52	I/II	Recruiting	NCT03661307
AML	Decitabine + clofarabine	727	II	Active, not recruiting	NCT02085408
AML	Decitabine + ruxolitinib Phosphate	42	I/II	Recruiting	NCT02257138
AML	Decitabine + bortezomib	165	II	Active, not recruiting	NCT01420926
AML	Decitabine + cytarabine + daunorubicin hydrochloride	180	II	Active, not recruiting	NCT01627041
*Guadecitabine (SGI-110)-based trials*					
AML	Guadecitabine	815	III	Completed	NCT02348489
Philadelphia-negative MDS	Guadecitabine	50	II	Recruiting	NCT03075826
High-risk MDS	Guadecitabine	103	II	Recruiting	NCT02131597
Advanced hepatocellular carcinoma (HCC)	Guadecitabine	51	II	Completed	NCT01752933
AML, MDS	Guadecitabine	401	I/II	Completed	NCT01261312
MDS, CMML	Guadecitabine	408	III	Recruiting	NCT02907359
AML, MDS	Guadecitabine + DLI	40	II	Not yet recruiting	NCT03454984
MDS relapsing post AlloSCT	Guadecitabine + DLI	90	II	Recruiting	NCT02684162
Refractory metastatic colorectal cancer	Guadecitabine + nivolumab	45	I/II	Not yet recruiting	NCT03576963
Recurrent ovarian, primary peritoneal, or fallopian tube cancer	Guadecitabine + Pembrolizumab	38	II	Recruiting	NCT02901899
Metastatic colorectal cancer	Guadecitabine + irinotecan	108	II	Active, not recruiting	NCT01896856
Advanced kidney cancer	Guadecitabine + durvalumab	58	I/II	Recruiting	NCT03308396
Refractory or resistant urothelial carcinoma	Guadecitabine + atezolizumab (anti-PD-L1 antibody)	53	II	Recruiting	NCT03179943
Advanced MDS CMML	Guadecitabine + atezolizumab	72	I/II	Recruiting	NCT02935361
Recurrent ovarian, fallopian tube, or primary peritoneal cancer	Guadecitabine + CDX-1401 Vaccine + atezolizumab	75	I/II	Recruiting	NCT03206047
Ovarian cancer	Guadecitabine + carboplatin	120	II	Completed	NCT01696032
*5-F-CdR-based trials*					
Advanced cancer	5-Fluoro-2-deoxycytidine (FdCyd)	58	I	Completed	NCT00359606
*Hydralazine-based trials*					
Ovarian cancer	Hydralazine + valproate	211	III	N/A	NCT00533299
Cervical cancer	Hydralazine + valproate	143	III	N/A	NCT00532818
Recurrent-persistent cervical cancer	Hydralazine + valproate	230	III	N/A	NCT02446652
Cervical cancer	Hydralazine + valproate + cisplatin	18	II	Completed	NCT00404326
Refractory solid tumors	Hydralazine + magnesium valproate	15	II	Completed	NCT00404508

Venetoclax, Bcl-2-selective inhibitor; Eltrombopag, c-mpl (TpoR) receptor agonist; APR-246, p53 agonist; DLI, donor leukocyte infusion; lenalidomide, derivative of thalidomide; sorafenib, multiple tyrosine kinase inhibitor; durvalumab, anti-PD-L1 monoclonal antibody; rigosertib, Ras mimetic; HAG regimen, homoharringtonine + cytarabine + G-CSF; lirilumab, anti-KIR monoclonal antibody; pembrolizumab, anti-PD-1 monoclonal antibody; lintuzumab, anti-CD33 monoclonal antibody; enasidenib, IDH2 inhibitor; avelumab, anti-PD-L1 monoclonal antibody; pevonedistat, NEDD8 inhibitor; nivolumab, anti-PD-1 monoclonal antibody; sirolimus, MTOR inhibitors; AG-120, IDH1 inhibitor; rituximab, anti-CD20 monoclonal antibody; PKC412, multitargeted protein kinase inhibitor; birinapant, SMAC mimetic antagonist; sonidegib, Hedgehog signaling pathway inhibitor; PF-04449913 (glasdegib), hedgehog signaling pathway inhibitor; etanercept, TNF inhibitor; ruxolitinib phosphate, JAK inhibitor; quizartinib, tyrosine kinase inhibitor; tosedostat, inhibitor of the M1 family of aminopeptidases; atezolizumab, anti-PD-L1 monoclonal antibody

### Inhibitors of histone modifications

Compared with DNA methylation, histone modifications have been investigated in broader areas of diseases, including solid tumors, hematological malignancies, and even many inflammatory diseases (such as viral infection, diabetes and inflammatory lung diseases). During the process of gene silencing, lysine deacetylation and demethylation of H3K4 rather than demethylation of H3K9 or cytosine methylation might be the primary causative event.^606^ Therefore, histone modification plays an essential role in the regulation of gene expression, which also makes it a promising target for disease treatment. Clinical trials targeting histone acetylation and histone methylation are listed in Table 6 and Table 7, respectively.

**Table 6:** Important ongoing clinical trials with histone acetylation-targeted therapies.

Condition	Design	Sample size	Phase	Current status	NCT
**Anti-HDAC**					
*Valproic acid-based trials*					
Advanced thyroid cancers	Valproic acid	13	II	Completed	NCT01182285
Uveal melanoma	Valproic acid	150	II	Recruiting	NCT02068586
Pancreatic cancer	Valproic acid	20	II	N/A	NCT01333631
Non-Hodgkin lymphoma, Hodgkin lymphoma, CLL	Valproic acid	52	II	N/A	NCT01016990
Locally advanced head and neck squamous cell carcinoma	Valproic acid + platinum-based chemoradiation	14	II	Completed	NCT01695122
Non-small-cell lung cancer	Valproic acid + chemoradiotherapy	20	I/II	N/A	NCT01203735
Recurrent high-grade glioma	Valproic acid + sildenafil citrate + sorafenib tosylate	66	II	Recruiting	NCT01817751
Glioma	Valproic acid + levetiracetam	120	IV	Recruiting	NCT03048084
Virus-associated cancer	Valproic acid + avelumab	39	II	Recruiting	NCT03357757
Colorectal cancer	Valproic acid + radiation therapy	152	I/II	N/A	NCT01898104
Refractory or relapsing small-cell lung cancer	Valproic acid + doxorubicin, cyclophosphamide and vindesine	64	II	Completed	NCT00759824
High-grade gliomas, brain tumors	Valproic acid + temozolomide + radiation therapy	43	II	Completed	NCT00302159
High-grade gliomas or diffuse intrinsic pontine glioma	Valproic acid + radiation	38	II	Active, not recruiting	NCT00879437
Advanced malignant neoplasm	Valproic acid + bevacizumab + temsirolimus	216	I	Recruiting	NCT01552434
Malignant mesothelioma	Valproic acid + doxorubicin	45	II	Completed	NCT00634205
Diffuse large B-cell lymphoma	Valproic acid + rituximab + CHOP	50	I/II	Completed	NCT01622439
*Sodium phenylbutyrate-based trials*					
Progressive or recurrent brain tumors	Phenylbutyrate	120	II	Completed	NCT00006450
Relapsed or refractory Epstein-Barr virus-positive cancer	Phenylbutyrate + valganciclovir	14	II	N/A	NCT00387530
Refractory or relapsed AML	Phenylbutyrate + dexamethasone + sargramostim	N/A	II	Completed	NCT00006240
*AN-9 (pivaloyloxymethyl butyrate)-based trials*					
Advanced non-small-cell lung cancer	Pivanex + docetaxel	225	II	Completed	NCT00073385
*Phenylacetate-based trials*					
Children with recurrent or progressive brain tumors	Phenylacetate	N/A	II		NCT00003241
*Vorinostat (SAHA)-based trials*					
Advanced cancer	Vorinostat	143	I	Active, not recruiting	NCT01266057
BRAFV600-mutated advanced melanoma	Vorinostat	22	I/II	Recruiting	NCT02836548
Breast cancer	Vorinostat	49	I/II	N/A	NCT00416130
Advanced, metastatic soft tissue sarcoma	Vorinostat	40	II	Completed	NCT00918489
AML	Vorinostat	37	II	Completed	NCT00305773
Advanced non-small-cell lung cancer	Vorinostat	16	II	Completed	NCT00138203
Recurrent or persistent ovarian epithelial or primary peritoneal cavity cancer	Vorinostat	60	II	Completed	NCT00132067
advanced adenoid cystic carcinoma advanced thyroid cancer	Vorinostat	30	II	Completed	NCT01175980
Advanced thyroid cancer	Vorinostat	19	II	Completed	NCT00134043
Kidney cancer	Vorinostat	14	II	Completed	NCT00278395
Metastatic or unresectable melanoma	Vorinostat	32	II	Completed	NCT00121225
Low-grade non-Hodgkin lymphoma	Vorinostat	37	II	Completed	NCT00253630
Progressive glioblastoma multiforme	Vorinostat	103	II	Completed	NCT00238303
Progressive metastatic prostate cancer	Vorinostat	29	II	Completed	NCT00330161
Advanced cutaneous T-cell lymphoma	Vorinostat	74	II	Completed	NCT00091559
Advanced malignant pleural mesothelioma	Vorinostat	662	III	Completed	NCT00128102
Metastatic or recurrent gastric cancer	Vorinostat + capecitabine + cisplatin	45	I/II	Completed	NCT01045538
Breast cancer	Vorinostat + tamoxifen	43	II	Completed	NCT00365599
T-cell non-Hodgkin lymphoma	Vorinostat + CHOP	14	I/II	Completed	NCT00787527
Advanced non-small-cell lung cancer	Vorinostat + bortezomib	18	II	Completed	NCT00798720
Relapsed or refractory multiple myeloma	Vorinostat + bortezomib	143	II	Completed	NCT00773838
Recurrent glioblastoma multiforme	Vorinostat + bortezomib	44	II	Completed	NCT00641706
Advanced soft tissue sarcoma	Vorinostat + bortezomib	16	II	Completed	NCT00937495
Multiple myeloma	Vorinostat + bortezomib	637	III	Completed	NCT00773747
Unresectable or metastatic kidney cancer	Vorinostat + bevacizumab	37	I/II	Completed	NCT00324870
Glioblastoma multiforme	Vorinostat + temozolomide + radiation therapy	125	I/II	Active, not recruiting	NCT00731731
Diffuse intrinsic pontine glioma	Vorinostat + radiation therapy	80	I/II	Active, not recruiting	NCT01189266
Recurrent ovarian cancer	vorinostat + paclitaxel + carboplatin vorinostat + pembrolizumab	70	II	N/A	NCT00772798
Stage IV non-small-cell lung cancer (NSCLC)	Vorinostat + pembrolizumab	100	I/II	Recruiting	NCT02638090
CLL, small lymphocytic lymphoma	Vorinostat + fludarabine phosphate + cyclophosphamide + rituximab	40	I/II	Active, not recruiting	NCT00918723
Relapse/refractory AML	Vorinostat + temozolomide	23	II	Completed	NCT01550224
Stage II, III, or IV diffuse large B-cell lymphoma	Vorinostat + rituximab	83	I/II	Active, not recruiting	NCT00972478
Metastatic breast cancer	Vorinostat + paclitaxel + bevacizumab	54	I/II	Completed	NCT00368875
High-grade glioma	Vorinostat + radiation therapy	101	II/III	Completed	NCT01236560
High-risk MDS, AML	Vorinostat + idarubicin + cytarabine	106	II	Completed	NCT00656617
Colorectal cancer	Vorinostat + hydroxychloroquine	76	II	Recruiting	NCT02316340
Advanced non-small-cell lung cancer	Vorinostat + carboplatin + paclitaxel	94	II	Completed	NCT00481078
Metastatic colorectal cancer	Vorinostat + fluorouracil + leucovorin calcium	58	II	Completed	NCT00942266
Recurrent glioblastoma multiforme (GBM)	Vorinostat + isotretinoin + temozolomide	135	I/II	Active, not recruiting	NCT00555399
Breast cancer	Vorinostat + carboplatin + nab-paclitaxel	68	II	Completed	NCT00616967
Diffuse large B-cell non-Hodgkin lymphoma	Vorinostat + chemotherapy + rituximab	107	I/II	Active, not recruiting	NCT01193842
Advanced sarcoma	Vorinostat + gemcitabine + docetaxel	67	I/II	Recruiting	NCT01879085
AML	Vorinostat + cytarabine + daunorubicin Hydrochloride/idarubicin	754	III	Completed	NCT01802333
Neuroblastoma	Vorinostat + 131I-MIBG	105	II	Recruiting	NCT02035137
Multiple myeloma	Vorinostat + lenalidomide	4420	III	Active, not recruiting	NCT01554852
Relapsed/refractory cutaneous T-cell lymphoma (CTCL)	Vorinostat vs KW-0761	372	III	Active, not recruiting	NCT01728805
*TSA (Trichostatin A)-based trials*					
Relapsed or refractory hematologic malignancies	Trichostatin A	42	I	Recruiting	NCT03838926
*Belinostat (PAHA, PXD101)-based trials*					
Advanced solid tumors or lymphoma	Belinostat	121	I	Completed	NCT00413075
Relapsed or refractory peripheral T-cell lymphoma	Belinostat	129	II	Completed	NCT00865969
Liver cancer	Belinostat	54	I/II	Completed	NCT00321594
MDS	Belinostat	21	II	Completed	NCT00357162
Relapsed or refractory aggressive B-cell non-Hodgkin lymphoma	Belinostat	22	II	Completed	NCT00303953
Advanced multiple myeloma	Belinostat	25	II	Completed	NCT00131261
Solid tumors or hematological malignancies	Belinostat + warfarin	27	I	Completed	NCT01317927
Soft tissue sarcomas	Belinostat + doxorubicin	41	I/II	Completed	NCT00878800
Relapsed/refractory NHL	Belinostat + carfilzomib	19	I	Completed	NCT02142530
Relapsed or refractory AML, MDS	Belinostat + pevonedistat	45	I	Not yet recruiting	NCT03772925
Adult T-cell leukemia-lymphoma	Belinostat + zidovudine	20	II	Recruiting	NCT02737046
Recurrent ovarian epithelial cancer	Belinostat + carboplatin	29	II	Completed	NCT00993616
Stage IV non-small-cell lung cancer (NSCLC)	Belinostat + carboplatin + paclitaxel	23	I/II	Completed	NCT01310244
Ovarian cancer	Belinostat + carboplatin + paclitaxel	80	I/II	Completed	NCT00421889
Cancer of unknown primary site	Belinostat + carboplatin + paclitaxel	89	II	Completed	NCT00873119
*Entinostat (MS-275)-based trials*					
Relapsed or refractory Hodgkin lymphoma	Entinostat	49	II	Completed	NCT00866333
MDS, AML, ALL	Entinostat	24	II	Completed	NCT00462605
Metastatic melanoma	Entinostat	28	II	Completed	NCT00185302
Advanced breast cancer	Entinostat	512	III	Recruiting	NCT03538171
Metastatic kidney cancer	Entinostat + aldesleukin	45	I/II	Active, not recruiting	NCT01038778
TN breast cancer	Entinostat + atezolizumab	88	I/II	Active, not recruiting	NCT02708680
Advanced epithelial ovarian cancer	Entinostat + avelumab	140	I/II	Active, not recruiting	NCT02915523
Metastatic colorectal cancer	Entinostat + regorafenib + hydroxychloroquine	44	I/II	Recruiting	NCT03215264
Advanced renal cell carcinoma	Entinostat + bevacizumab + atezolizumab	62	I/II	Recruiting	NCT03024437
Endometrioid endometrial cancer	Entinostat + medroxyprogesterone acetate	50	II	Active, not recruiting	NCT03018249
Renal cell carcinoma	Entinostat + IL-2	46	II	Recruiting	NCT03501381
NSCLC, melanoma, and colorectal cancer	Entinostat + pembrolizumab	202	I/II	Active, not recruiting	NCT02437136
Relapsed and refractory lymphomas	Entinostat + pembrolizumab	78	II	Recruiting	NCT03179930
Stage III/IV melanoma	Entinostat + pembrolizumab	14	II	Recruiting	NCT03765229
High-risk refractory malignancies	Entinostat + nivolumab	128	I/II	Not yet recruiting	NCT03838042
Metastatic cholangiocarcinoma and pancreatic adenocarcinoma	Entinostat + nivolumab	54	II	Recruiting	NCT03250273
Renal cell carcinoma	Entinostat + nivolumab + ipilimumab	53	II	Recruiting	NCT03552380
Advanced breast cancer	Entinostat + exemestane	130	II	Completed	NCT00676663
Breast cancer	Entinostat + exemestane	600	III	Active, not recruiting	NCT02115282
Advanced NSCLC	Entinostat + erlotinib	132	I/II	Completed	NCT00602030
Non-small-cell lung carcinoma	Entinostat + erlotinib	70	II	Completed	NCT00750698
*Panobinostat (LBH589)-based trials*					
High-risk MDS, AML	Panobinostat	62	I/II	Active, not recruiting	NCT01451268
Advanced hematological malignancies	Panobinostat	175	I/II	Completed	NCT00621244
Metastatic thyroid cancer	Panobinostat	13	II	Completed	NCT01013597
Advanced soft tissue sarcoma	Panobinostat	53	II	Completed	NCT01136499
Refractory prostate cancer	Panobinostat	35	II	Completed	NCT00667862
Refractory clear cell renal carcinoma	Panobinostat	20	II	Completed	NCT00550277
Relapsed/refractory classical Hodgkin lymphoma	Panobinostat	129	II	Completed	NCT00742027
Refractory colorectal cancer	Panobinostat	29	II	Completed	NCT00690677
HER2-negative locally recurrent or metastatic breast cancer	Panobinostat	54	II	Completed	NCT00777049
Relapsed and bortezomib-refractory multiple myeloma	Panobinostat	55	II	Completed	NCT01083602
Relapsed or refractory non-Hodgkin lymphoma	Panobinostat	41	II	Active, not recruiting	NCT01261247
Refractory CML	Panobinostat	27	II/III	Completed	NCT00449761
Refractory/resistant cutaneous T-cell lymphoma	Panobinostat	9	II/III	Completed	NCT00490776
Refractory CML	Panobinostat	29	II/III	Completed	NCT00451035
Refractory cutaneous T-cell lymphoma	Panobinostat	139	II/III	Completed	NCT00425555
Hodgkin lymphoma (HL)	Panobinostat	41	III	Completed	NCT01034163
Relapsed/refractory multiple myeloma	Panobinostat + carfilzomib	80	I/II	Active, not recruiting	NCT01496118
Recurrent high-grade glioma	Panobinostat + bevacizumab	51	I/II	Completed	NCT00859222
Recurrent prostate cancer after castration	Panobinostat + bicalutamide	52	I/II	Completed	NCT00878436
AML	Panobinostat + idarubicin + cytarabine	46	I/II	Completed	NCT00840346
Diffuse large B-cell lymphoma (DLBCL)	Panobinostat + rituximab	42	II	N/A	NCT01238692
Relapsed and refractory lymphoma	Panobinostat + everolimus	31	I/II	Completed	NCT00967044
Gliomas	Panobinostat + everolimus	32	II	Recruiting	NCT03632317
Recurrent multiple myeloma, Non-Hodgkin lymphoma, or Hodgkin lymphoma	Panobinostat + everolimus	124	I/II	Active, not recruiting	NCT00918333
Relapsed/refractory peripheral T-cell lymphoma or NK/T-cell lymphoma	Panobinostat + bortezomib	25	II	Completed	NCT00901147
Relapsed or relapsed- and-refractory multiple myeloma	Panobinostat + bortezomib + dexamethasone	240	II	Recruiting	NCT02654990
Relapsed multiple myeloma	Panobinostat + bortezomib + dexamethasone	768	III	Completed	NCT01023308
Relapsed or refractory Hodgkin lymphoma	Panobinostat + lenalidomide	24	II	Completed	NCT01460940
*Mocetinostat (MGCD0103)-based trials*					
Advanced solid tumors or non-Hodgkin lymphoma	Mocetinostat	42	I	Completed	NCT00323934
Refractory chronic lymphocytic leukemia	Mocetinostat	21	II	Completed	NCT00431873
Relapsed and refractory lymphoma	Mocetinostat	74	II	Completed	NCT00359086
Tumors	Mocetinostat + gemcitabine	47	I/II	Completed	NCT00372437
Relapsed or refractory Hodgkin lymphoma	Mocetinostat + brentuximab vedotin	7	I/II	Active, not recruiting	NCT02429375
Advanced solid tumors and NSCLC	Mocetinostat + durvalumab	119	I/II	Active, not recruiting	NCT02805660
Metastatic leiomyosarcoma	Mocetinostat + gemcitabine	20	II	Completed	NCT02303262
Non-small-cell lung cancer	Mocetinostat + glesatinib + sitravatinib + nivolumab	209	II	Recruiting	NCT02954991
*CI-994-based trials*					
Advanced myeloma	CI-994	6	II	Completed	NCT00005624
Advanced pancreatic cancer	CI-994 + gemcitabine	N/A	II	Completed	NCT00004861
Advanced non-small-cell lung cancer	CI-994 + gemcitabine	N/A	III	Completed	NCT00005093
*Romidepsin (Depsipeptide, FR901228, FK228)-based trials*					
Recurrent high-grade gliomas	Romidepsin	50	I/II	Completed	NCT00085540
Progressive or relapsed peripheral T-cell lymphoma	Romidepsin	131	II	Active, not recruiting	NCT00426764
Soft tissue sarcoma	Romidepsin	40	II	Completed	NCT00112463
Squamous cell carcinoma of the head and neck	Romidepsin	14	II	Completed	NCT00084682
Metastatic breast cancer	Romidepsin	37	II	Completed	NCT00098397
Relapsed small-cell lung cancer	Romidepsin	36	II	Completed	NCT00086827
Cutaneous T-cell lymphoma and peripheral T-cell lymphoma	Romidepsin	131	II	Completed	NCT00007345
Relapsed or refractory AML	Romidepsin	47	II	Completed	NCT00062075
Relapsed or refractory multiple myeloma	Romidepsin	50	II	Completed	NCT00066638
Relapsed or refractory non-Hodgkin lymphoma	Romidepsin	35	II	Completed	NCT00077194
Triple-negative breast cancer (TNBC)	Romidepsin + nivolumab + cisplatin	54	I/II	Recruiting	NCT02393794
Relapsed/refractory T-cell lymphoma	Romidepsin + tenalisib	42	I/II	Recruiting	NCT03770000
Lymphoid hematopoietic malignancy	Romidepsin + pembrolizumab	39	I/II	Recruiting	NCT03278782
Peripheral T-cell lymphoma (PTCL)	Romidepsin + ixazomib	48	I/II	Recruiting	NCT03547700
Relapsed/refractory lymphoid malignancies	Romidepsin + pralatrexate	93	I/II	Recruiting	NCT01947140
Peripheral T-cell lymphoma	Romidepsin + CHOP	421	III	Active, not recruiting	NCT01796002
Relapsed/refractory peripheral T-cell lymphoma	Romidepsin + gemcitabine	20	II	Completed	NCT01822886
Relapsed or refractory lymphomas and myeloma	Romidepsin + lenalidomide	62	I/II	Active, not recruiting	NCT01755975
Relapsed or refractory B- and T-cell lymphomas	Romidepsin + lenalidomide + carfilzomib	31	I/II	Active, not recruiting	NCT02341014
Peripheral T-cell lymphoma	Romidepsin + lenalidomide	35	II	Recruiting	NCT02232516
*Nicotinamide-based trials*					
Skin cancer prevention	Nicotinamide	120	II	Recruiting	NCT03769285
Lung cancer	Nicotinamide	110	II/III	Active, not recruiting	NCT02416739
Bladder cancer	Niacinamide + radiation + carbogen	330	III	Completed	NCT00033436
**Inhibitors of sirtuins**					
*Suramin-based trials*					
Recurrent primary brain tumors	Suramin	N/A	II	Completed	NCT00002639
Hormone-refractory prostate cancer	Suramin	390	III	Completed	NCT00002723
Metastatic renal cell (kidney) cancer	Suramin + fluorouracil	36	I/II	Completed	NCT00083109
Advanced non-small-cell lung cancer	Suramin + docetaxel	80	II	N/A	NCT01671332
Stage IIIB-IV breast cancer	Suramin + paclitaxel	31	I/II	Completed	NCT00054028
Stage IIIB or IV non-small-cell lung cancer	Suramin + paclitaxel + carboplatin	82	II	Completed	NCT00006929
Poor-prognosis prostate carcinoma	Suramin + flutamide + leuprolide	70	II	Completed	NCT00001266
Prostate cancer	Suramin + flutamide + hydrocortisone	N/A	III	Completed	NCT00002881
**Inhibitors for HATs**					
*CBP-targeted therapy*					
Advanced myeloid malignancies	PRI-724	49	I/II	Completed	NCT01606579
Advanced pancreatic adenocarcinoma	PRI-724 + gemcitabine	20	I	Completed	NCT01764477
**BRD (BET) inhibitors**					
*GSK525762 (I-BET762, molibresib)-based trials*					
Relapsed, refractory hematologic malignancies	GSK525762	180	I	Recruiting	NCT01943851
NUT midline carcinoma (NMC) and other cancers	GSK525762	195	I	Active, not recruiting	NCT01587703
Castration-resistant prostate cancer	GSK525762 + androgen deprivation therapy	37	I	Active, not recruiting	NCT03150056
Advanced or metastatic breast cancer	GSK525762 + fulvestrant	294	II	Recruiting	NCT02964507
*CPI-0610-based trials*					
Multiple myeloma	CPI-0610	30	I	Completed	NCT02157636
Progressive lymphoma	CPI-0610	64	I	Active, not recruiting	NCT01949883
*RO6870810 (TEN-010, RG6146, JQ2)-based trials*					
AML, MDS	RO6870810	26	I	Completed	NCT02308761
Advanced solid tumors	RO6870810	52	I	Completed	NCT01987362
Advanced multiple myeloma	RO6870810	86	I	Recruiting	NCT03068351
Advanced ovarian cancer or triple-negative breast cancer	RO6870810 + atezolizumab	116	I	Suspended	NCT03292172
High-grade B-cell lymphoma	RO6870810 + venetoclax + rituximab	94	I	Recruiting	NCT03255096
*BAY1238097-based trials*					
Neoplasms	BAY1238097	8	I	Terminated	NCT02369029
*MK8628 (OTX-015, birabresib)-based trials*					
Advanced solid tumors	MK-8628	47	I	Completed	NCT02259114
Hematologic malignancies	MK-8628	9	I	Active, not recruiting	NCT02698189
Hematologic malignancies	MK-8628	141	I	Completed	NCT01713582
*FT-1101-based trials*					
Relapsed or refractory hematologic malignancies	FT-1101	160	I	Recruiting	NCT02543879
*INCB057643-based trials*					
Advanced malignancies	INCB057643	136	I/II	Active, not recruiting	NCT02711137

Lenalidomide, derivative of thalidomide; durvalumab, anti-PD-L1 monoclonal antibody; avelumab, anti-PD-L1 monoclonal antibody; bevacizumab, VEGF inhibitor; temsirolimus, mTOR inhibitor; rituximab, anti-CD20 monoclonal antibody; regorafenib, multikinase inhibitor; nivolumab, anti-PD-1 monoclonal antibody; sitravatinib, multiple tyrosine kinase inhibitor; tenalisib, inhibitor of PI3K; pembrolizumab, anti-PD-1 monoclonal antibody; atezolizumab, anti-PD-L1 monoclonal antibody

**Table 7:** Important ongoing clinical trials with histone methylation-targeted therapies.

Condition	Design	Sample size	Phase	Current status	NCT
**HMT inhibitor**					
DOT1L-targeted therapy					
*Pinometostat (EPZ-5676)-based trials*					
Relapsed/refractory leukemias	Pinometostat	51	I	Completed	NCT01684150
Acute myeloid leukemia with MLL gene rearrangement	Pinometostat + standard chemotherapy	37	I/II	Recruiting	NCT03724084
*Tazemetostat (EPZ-6438)-based trials*					
Diffuse large B-cell lymphoma	Tazemetostat	133	I/II	Suspended	NCT02889523
Advanced tumors/lymphomas	Tazemetostat	420	I/II	Recruiting	NCT01897571
Relapsed or refractory B-cell lymphoma with EZH2 gene mutation	Tazemetostat	21	II	Active, not recruiting	NCT03456726
Recurrent ovarian, primary peritoneal, or endometrial cancer	Tazemetostat	43	II	Recruiting	NCT03348631
Lymphoma, advanced solid tumors	Tazemetostat	300	II	Recruiting	NCT02875548
Malignant mesothelioma	Tazemetostat	67	II	Active, not recruiting	NCT02860286
INI1-negative tumors or relapsed/ refractory synovial sarcoma	Tazemetostat	250	II	Recruiting	NCT02601950
Relapsed/refractory lymphoma	Tazemetostat + atezolizumab	92	I	Active, not recruiting	NCT02220842
Advanced urothelial carcinoma	Tazemetostat + pembrolizumab	30	I/II	Not yet recruiting	NCT03854474
*GSK2816126-based trials*					
Relapsed/refractory lymphomas, solid tumors and multiple myeloma	GSK2816126	41	I	Terminated	NCT02082977
*CPI-1205-based trials*					
B-cell lymphomas	CPI-1205	41	I	Active, not recruiting	NCT02395601
Advanced solid tumors	CPI-1205	24	I/II	Active, not recruiting	NCT03525795
Castration-resistant prostate cancer	CPI-1205	242	I/II	Recruiting	NCT03480646
**Histone demethylase inhibitors**					
LSD1-targeted therapy					
*TCP-based trials*					
AML and MDS	TCP	17	I	Active, not recruiting	NCT02273102
Relapsed or refractory AML	TCP	16	I/II	N/A	NCT02261779
*ORY-2001-based trials*					
Mild to moderate Alzheimer's disease	ORY-2001	33	II	Not yet recruiting	NCT03867253
*GSK2879552-based trials*					
High-risk MDS	GSK2879552 + azacitidine	74	II	Recruiting	NCT02929498
*4SC-202-based trials*					
Advanced hematologic malignancy	4SC-202	36	I	Completed	NCT01344707
Malignant melanoma	4SC-202 + pembrolizumab	40	I/II	Recruiting	NCT03278665

Atezolizumab, anti-PD-L1 monoclonal antibody; pembrolizumab, anti-PD-1 monoclonal antibody

#### Inhibitors for HATs and BETs

Generally, there are two strategies for preventing aberrant histone acetylation, including altering interactions within the active sites within HATs or using mimetic products of enzymatic substrates. To date, many inhibitors targeting BRD proteins have been investigated in clinical trials, whereas there are no clinical trials investigating inhibitors for HATs.

Bisubstrate inhibitors are selective inhibitors for PCAF, p300, and TIP60. They mimic two substrates of HATs: the cofactor acetyl coenzyme A (Ac-CoA) and a peptide resembling the lysine substrate.^607,608^ However, due to their peptidic nature and size, they are not membrane-permeable and require the assistance of a delivery system. Based on inhibitory strategies for HATs, nonpeptide small molecular inhibitors have been developing as potential therapeutic agents. Several small molecule inhibitors are natural products, including garcinol, curcumin, and anacardic acid.^609–611^ These natural HAT inhibitors lack selectivity between HATs and often have other targets. Therefore, structurally modified and synthetic compounds have been reported. Α-Methylene-g-butyrolactones are small molecular inhibitors of HATs with selectivity for either GCN5L2 or PCAF.^612^ Isothiazolone is another HAT inhibitor targeting p300 and PCAF.^613^ However, high reactivity towards thiolates limits the application of HAT inhibitors in biological systems. Other inhibitors of HATs, such as thiazide sulfonamide and C646, have been gradually identified and show promising effects in multiple cancers. Another strategy to inhibit HAT activity is to target protein–protein interactions between HATs and their interaction partners. This method is dependent on the function of the interactions rather than the acetylation activity of HATs. ICG-001 and PRI-724 are representatives of this kind of inhibitor. Appropriately applying HAT agonists is also important to correct aberrant acetylation during diseases. CTPB is derived from anacardic acid and selectively activates p300, resulting in gene transcription.^609^ TTK21 and SPV106 are two other agonists based on anacardic acid.

Binding to BRDs and blocking acetylated lysine recognition is another mechanism that inhibits acetylation. JQ1 and I-BET762 are two representative inhibitors of the BET family. JQ1 is a cell-permeable small molecule and can competitively bind to BRD4 fusion oncoproteins, such as BRD4-NUT, resulting in cancer cell differentiation and apoptosis.^614^ Similarly, I-BET762 is also a synthetic mimic of and competes with BRD4.^615^ Other compounds, such as MS417, OTX-015, RVX-208, OXFBD, I-BET151, PFI-1, MS436, and XD14, are also BET inhibitors and have been well illustrated in other published papers.^616^ We will focus on the associations between these compounds and cancers. However, a number of non-BET proteins containing BRDs have attracted considerable attention. Many non-BET bromodomain inhibitors are based on a structure called the “WPF shelf” and a “gatekeeper” residue located at the start of the C helix.^617^ Several HATs have a BRD, such as Gcn5, PCFA, p300, and CBP. Inhibitors for CBP include MS2126, MS7972, ischemin, SGC-CBP30 and I-CBP112; optimized 1-(1H-indol-1-yl) ethanone derivatives have also shown promising results in inhibiting CBP and p300.^618^ BAZ2A/B bromodomain inhibitors include BAZ2-ICR and GSK2801. The quinolone-fused lactam LP99 was the first synthetic selective inhibitor for BRD7/9. I-BRD9 was identified by GlaxoSmithKline (GSK) and is a selective inhibitor of BRD9, which has more than 200-fold selectivity for BRD9 over BRD7 and 700-fold selectivity for BRD9 over BET family members.^619^ PFI-3 is a potential inhibitor of SMARCA4 and PB1 with a stronger affinity for the bromodomain of SMARCA4. However, Vangamudi et al. identified that the ATPase domain within SMARC4 bypassed the anticancer effects related to the bromodomain since PFI-3 did not inhibit cell proliferation.^620^ The BRPF1 (bromodomain and PHD finger-containing 1) protein is part of the BRPF family, which is a component of MYST family complexes. The inhibitors of BRPF1 include PFI-4, OF-1, and NI-57. 1,3-Dimethyl benzimidazolones were the first selective inhibitors of BRPF1. PFI-4 and OF-1 are two close analogs of 1,3-dimethyl benzimidazolone that have been identified by the Structural Genomics Consortium (SGC). Another BRPF1 inhibitor, NI-57, was discovered by the SGC based on a new quinolinone scaffold. Both NI-57 and OF-1 are thought to interact with BRPF1-3 as pan-BRPF bromodomain inhibitors. Based on the bromodomain contained within both TRIM24 (tripartite motif containing protein 24) and BRPF1, a dual inhibitor, IACS-9571, has been identified.^621^ Bromosporine is a panbromodomain inhibitor with good cellular activity, whereas in a recent study, researchers noticed that bromodomain inhibitors only targeted the BET family rather than other BRDs.^622^

#### Inhibition of HDACs

Given that multiple methods can regulate HDAC activity, the designation of HDAC inhibitors has its own advantages. In the 1970s, butyrate was found to induce the accumulation of acetylated histones in cancer cells, which is thought to be associated with the inhibition of deacetylation.^623^ Later, a natural extract, trichostatin A (TSA), was identified to inhibit the activity of partially purified HDACs and induce cancer cell differentiation and apoptosis.^624^ Gradually, more natural and synthetic compounds have been identified to inhibit histone deacetylation. A study reported that administration of HDAC inhibitors only regulates a small number of genes (1–2%) but induces an obvious and rapid decrease in *c-Myc* gene expression, which indicated that a restricted set of cellular genes was uniquely sensitive to regulation of histone acetylation.^625^ The combination of two HDAC inhibitors, SAHA and TSA, induced melanoma cell growth arrest by upregulating p21, p27 and NF-κB, and MG132 can enhance the effect of TSA.^626^ The inhibition of HDACs has been investigated in various cancers, with promising antitumor effects.^627,628^ Based on the characteristics of their chemical structures, HDAC inhibitors can be divided into five groups: short-chain fatty acids, hydroxamic acids, benzamides, cyclic peptides, and hybrid molecules. In addition to those included in the five groups, some new synthetic compounds also act as inhibitors of HDACs.

The short-chain fatty acid group contains sodium butyrate, valproic acid (VPA), sodium phenylbutyrate, and AN-9 (pivaloyloxymethyl butyrate). The effective concentration of butyrate is usually at the micromolar level. The group of hydroxamic acids includes more than ten members and is the best-studied class. Structural analyses of TSA and suberoylanilide hydroxamic acid (SAHA) show that they are noncompetitive inhibitors of HDACs since they share significant homology with class I and class II HDACs, which makes them mimics of the lysine substrates.^629^ In addition, they chelate the active zinc ion in a bidentate manner, which is crucial for enzymatic activity.^624^ Hexamethylene bisacetamide (HMBA) is a representative of the hybrid polar compounds (HPCs), whereas second-generation HPCs, such as oxamflatin, SAHA, suberic bishydroxamic acid (SBHA), and m-carboxycinnamic acid bishydroxamide (CBHA), have shown better inhibition of HDACs and anticancer effects than first-generation agents.^630^ Oxamflatin, scriptaid, and amide are analogs of TSA and show anticancer effects.^631–633^ Benzamide inhibitors (MS-275, MGCD0103, and CI-994) are well-studied and show promising effects in the treatment of diseases, especially cancers. They inhibit histone deacetylation via binding to catalytic zinc ions within HDACs through carbonyl and amino groups. Inhibition of HDACs by benzamide inhibitors is thought to be reversible, but the bond may become tight and pseudoirreversible in a time-dependent manner.^634,635^ However, benzamide inhibitors have less activity than members of the hydroxamate or cyclic peptide families, with an effective concentration around the micromolar range.^636^ Cyclic peptides can be further divided into two groups: cyclic tetrapeptide containing a 2-amino-8-oxo-9, 10-epoxy-decanoyl (AOE) moiety (HC-toxin, trapoxin) and cyclic peptides without the AOE moiety (apicidin and romidepsin). The epoxyketone group is essential for the inhibitors to bind to active zinc ions, but the epoxyketone-based bond is irreversible. Trapoxin is a fungal cyclic peptide and can irreversibly inhibit the activity of HDACs.^637^ Romidepsin, also known as FK228, most likely relies on one of the thiol groups to coordinate to the active site zinc ion.^638^ Garlic-associated derivatives, such as diallylsulfide and allylmercaptan, are capable of generating a thiol group that makes them potential inhibitors of HDACs.^639^ K-trap, an analog of trapoxin, and other derivatives, including 9-acyloxyapicidins and 9-hydroxy, have been under investigation. Depudecin is a natural epoxide derivative isolated from the fungus *Alternaria brassicicola*. Psammaplins is isolated from a marine sponge *Pseudoceratina purpurea*. These two natural extracts can inhibit the activity of HDACs.

Early HDAC inhibitors were nonselective because of the high homology of the structure and catalytic mechanism of HDACs within each group. The first selective HDAC inhibitor was tubacin, which targets HDAC6 with increased tubulin acetylation but not histone acetylation.^640^ PCI-34051, a specific inhibitor of HDAC8, can induce caspase-dependent apoptosis in T-cell lymphoma but does not increase histone acetylation.^641^ Another benzamide inhibitor, SHI-1:2, shows HDAC1/HDAC2-specific inhibitory activity that is >100-fold more selective than that of other HDACs.^642^ New synthetic chemicals, such as SK7041 and splitomicin, selectively target class I HDACs and sir2-like family members, respectively. The same efforts have been made to develop inhibitors for sirtuins, the class III HDACs. Nicotinamide, a byproduct of the sirtuin enzyme reaction, is a widely used inhibitor of all sirtuins. Other compounds, such as cambinol, salermide, tenovin, EX-527, suramin, and AGK2, have also been reported as sirtuin inhibitors. Sirtuin inhibitors (such as nicotinamide) function via interactions with the NAD+ within the active site of sirtuins or through binding to acetyl-lysine.

Of note, second-generation HDACs, including hydroxamic acids (vorinostat (SAHA), belinostat (PXD101), LAQ824, and panobinostat (LBH589)) and benzamides (entinostat (MS-275), tacedinaline (CI-994), and mocetinostat (MGCD0103)), are currently in clinical trials, and some of them have already been approved for disease treatment. The success of romidepsin in phase I clinical trials in cutaneous and peripheral T-cell lymphoma accelerated the development of HDAC inhibitors as anticancer drugs. In 2006, SAHA (vorinostat) was first approved by the US Food and Drug Administration (FDA) for the treatment of cancer, restricted to patients with cutaneous T-cell lymphoma (CTCL), as an HDAC inhibitor.^643^ Romidepsin (Istodax) was the second approved HDAC inhibitor, which was approved in 2009. Three members of the benzamide family have also shown clinical significance in anticancer drug development. Belinostat (Beleodaq, previously known as PXD101) was approved in 2014 by the US FDA and European Medicines Agency to treat peripheral T-cell lymphoma. Another HDAC inhibitor, panobinostat, is a nonselective HDAC (pan-HDAC). It has shown promising effects in anticancer treatments; therefore, the FDA accelerated its approval for the treatment of patients with multiple myeloma. Intriguing, as we mentioned before, truncating mutations in HDAC2 have been found in sporadic carcinomas and colorectal cancer and result in resistance to traditional HDAC inhibitors.^644^ Mutations in other HDACs also exist; therefore, screening of these mutations in cancer can improve the efficacy of HDAC inhibitors.

#### Inhibitor of HMTs and HDMTs

EPZ004777 was the first identified selective inhibitor of DOT1L and selectively kills MLL-translocated cells over those without MLL translocation.^645^ However, due to its poor pharmacokinetic properties, a second generation of EPZ004777, EPZ-5767, was developed with a cyclobutyl ring replacing the ribose moiety.^646^ EPZ-5767 also shows synergistic effects with cytarabine, daunorubicin, and the DNMT inhibitor azacitidine in treatments for ALL with MLL translocation. EPZ-5767, though still showing low oral bioavailability, has been investigated in clinical trials for the treatment of leukemia with MLL rearrangement.^647^ There are several inhibitors of EZH2. 3-Deazaneplanocin A (DZNep), a derivative of the antibiotic neplanocin-A, is one of the most studied compounds. In fact, DZNep is a SAH-hydrolase inhibitor and decreases EZH2 expression via upregulation of SAH, which leads to degradation of PRC2 in a feedback inhibition mechanism.^648,649^ Another kind of inhibitor is SAM competitive inhibitors. SAM is responsible for the methyl moiety of KMTs. EI1, a small molecular inhibitor of EZH2, inhibits EZH2 activity by directly binding to EZH2 and competing with SAM.^650^ GSK343 and GSK126 are two other SAM competitive inhibitors that have been investigated in clinical trials.^651,652^ EPZ005687, a potent inhibitor of EZH2, significantly reduces H3K27 methylation in lymphoma cells with point mutations at the Tyr641 and Ala677 residues of EZH2 without obvious effects on the proliferation of wild-type cells.^653^ EPZ-6438, which shows similar effects and superior oral bioavailability, was developed next.^654^ CPI-1205 is a novel inhibitor of EZH2 that belongs to the pyridone family.

Tranylcypromine (TCP) is an approved drug for depression due to its ability to inhibit monoamine oxidase (MAO) activity. The structures of LSD enzymes and MAOs share many similarities. Therefore, the side effects of TCP as an HDMT inhibitor, including orthostatic hypotension, dizziness, and drowsiness,^655^ are mostly caused by targeting of MAO. Administration of TCP in MLL-AF9 leukemia promotes tumor cell differentiation and apoptosis.^656^ TCP is also capable of resensitizing non-acute promyelocytic leukemia (APL) AML cells to all-trans retinoic acid (ATRA) treatment via increasing H3K4me2 and the expression of myeloid-differentiation-associated genes.^657^ Several derivatives of TCP have been developed to achieve better bioavailability and selectivity, including OG-002, RN-1, SP2509, and GSK690.^658–660^ Another LSD1 selective inhibitor, ORY-1001, can also promote the differentiation of leukemia cell lines, especially cells with translocations in MLL, and has good oral bioavailability.^661^ To date, three LSD1 inhibitors, including TCP, ORY-1001, and GSK2879552, have been under investigation in clinical trials for the treatment of cancer patients. Daminozide (N-(dimethylamino) succinamic acid, 160 Da), a plant growth regulator, selectively inhibits KDM2/7 by chelating the active site metal.^662^ Daminozide and siRNA can similarly downregulate KDM7 expression and then regulate tumor-repopulating cells via demethylation of H3K9.^663^ GSK-J1 was the first identified KDM6 inhibitor with restricted cellular permeability, which resulted from its highly polar structure. Its ethyl ester, GSK-J4, possesses an improved ability to enter cells.^664^ However, scientists have found that GSK-J1 shows compatible selectivity for the KDM6 and KDM5 families and that GSK-J4 is also a potential inhibitor for KDM5B and KDM4C.^665^ EPT-103182, a selective inhibitor of KDM5B, has shown promising results in terms of antiproliferative effects in hematological and solid cancer cells. KDM8 and JMJD6 share homology and can be inhibited by a broad spectrum inhibitor, NOG.^661^

Specific inhibitors usually have similar selectivity to closely related homologs within a group, and even across different groups, which needs to be taken into consideration when using compounds that are not highly selective.

### Combined therapy

Epigenetic regulation during tumorigenesis is complicated and involves multiple steps. Therefore, the combination of two or more therapies targeting various epigenetic events seems helpful. This combination synergistically inhibits the expression of tumor-growth-promoting genes and promotes the reexpression of tumor suppressor genes. 4SC-202 is a small molecular drug with dual effects that can inhibit HDAC1/2/3 and LSD1 with similar low micromolar potency. This drug is under clinical investigation. Other studies have administered two or more kinds of epigenetic drugs for anticancer therapy. Relevant clinical trials are listed in Table 8.

**Table 8:** Important ongoing clinical trials with combination therapies including DNA methylation and histone modification.

Condition	Design	Sample size	Phase	Current status	NCT
*Histone acetylation inhibitor* + *DNA methylation inhibitor*					
Solid tumors, hematologic malignancies	Azacitidine + pracinostat	85	I	Completed	NCT00741234
MDS	Azacitidine + pracinostat	102	II	Completed	NCT01873703
High-risk MDS	Azacitidine + pracinostat	60	II	Active, not recruiting	NCT03151304
AML	Azacitidine + pracinostat	500	III	Recruiting	NCT03151408
MDS	Azacitidine + mocetinostat	18	I/II	Completed	NCT02018926
High-risk MDS, AML	Azacitidine + mocetinostat	66	I/II	Completed	NCT00324220
Advanced cancers	Azacitidine + valproic acid	69	I	Completed	NCT00496444
AML, MDS	Azacitidine + valproic acid	50	II	Recruiting	NCT02124174
Intermediate II and high-risk MDS	Azacitidine + valproic acid	62	II	Completed	NCT00439673
AML, MDS	Azacitidine + valproic acid + ATRA	34	II	Completed	NCT00326170
High-risk MDS	Azacitidine + valproic acid/lenalidomide/idarubicin	320	II	Active, not recruiting	NCT01342692
Higher-risk MDS, CML	Azacitidine + vorinostat	282	II	Active, not recruiting	NCT01522976
AML, high-risk MDS	Azacitidine + vorinostat	260	II	Active, not recruiting	NCT01617226
AML, MDS	Azacitidine + vorinostat	135	I/II	Active, not recruiting	NCT00392353
Relapsed/refractory lymphoma	Azacitidine + vorinostat	17	I/II	Completed	NCT01120834
Relapsed/refractory lymphoid malignancies	Azacitidine + romidepsin	60	I/II	Recruiting	NCT01998035
Relapsed or refractory AITL	Azacitidine + romidepsin + bendamustine + gemcitabine	86	III	Recruiting	NCT03593018
Lymphoma	Azacitidine + romidepsin + durvalumab + pralatrexate	148	I/II	Recruiting	NCT03161223
Advanced non-small-cell lung cancer	Azacitidine + entinostat	162	II	Completed	NCT00387465
AML	Azacitidine + entinostat	108	II	Recruiting	NCT01305499
Advanced breast cancer	Azacitidine + entinostat	58	II	Active, not recruiting	NCT01349959
AML, MDS, CML	Azacitidine + entinostat	197	II	Completed	NCT00313586
Metastatic colorectal cancer	Azacitidine + entinostat	47	II	Completed	NCT01105377
Non-small-cell lung cancer	Azacitidine + entinostat + nivolumab	120	II	Recruiting	NCT01928576
Leukemia, lung cancer, lymphoma, multiple myeloma, prostate cancer	Azacitidine + phenylbutyrate	N/A	II	Completed	NCT00006019
AML with 11q23 rearrangement	Azacitidine + pinometostat	36	I/II	Not yet recruiting	NCT03701295
High-risk MDS	Azacitidine + GSK2879552	74	II	Recruiting	NCT02929498
AML, MDS	Decitabine + valproic acid	153	II	Completed	NCT00414310
Relapsed/refractory MDS, leukemia	Decitabine + valproic acid	54	I/II	Completed	NCT00075010
AML	Decitabine + valproic acid	204	II	Completed	NCT00867672
AML, MDS	Decitabine + vorinostat	71	I	Completed	NCT00479232
AML, MDS	Decitabine + panobinostat	52	I/II	Completed	NCT00691938
Relapsed or refractory leukemia and MDS	Decitabine + romidepsin	36	I	Completed	NCT00114257
Advanced lung cancer	Guadecitabine + mocetinostat + pembrolizumab	40	I	Recruiting	NCT03220477
Lung cancer	Hydralazine + valproic acid	29	I	Completed	NCT00996060
Metastatic cervical cancer	Hydralazine + valproate	143	III	N/A	NCT00532818
Ovarian cancer	Hydralazine + valproate	211	III	N/A	NCT00533299
Cervical cancer	Hydralazine + valproate + cisplatin chemoradiation	18	II	Completed	NCT00404326
Refractory solid tumors	Hydralazine + magnesium valproate	15	II	Completed	NCT00404508
*BET inhibitor* + *DNA methylation inhibitor*					
Relapsed or refractory hematologic malignancies	FT-1101 + azacitidine	160	I	Recruiting	NCT02543879
AML, MDS	GSK3326595 (selective inhibitor of protein arginine methyltransferase 5 (PRMT5)) vs azacitidine	302	I/II	Recruiting	NCT03614728

Pembrolizumab, anti-PD-1 monoclonal antibody; lenalidomide, derivative of thalidomide; durvalumab, anti-PD-L1 monoclonal antibody; nivolumab, anti-PD-1 monoclonal antibody

## Conclusion

Although more specific mechanisms need to be investigated, it is well accepted that epigenetic events are important in normal biological processes as well as in tumorigenesis and that the epigenetic status is usually widely altered during cancer initiation. This makes epigenome-targeted therapy a promising strategy for the treatment of cancer. Based on the complexity of cancer, epigenetic alterations have influenced multiple aspects in cancer, such as the expression of oncogenes and tumor suppressor genes and signal transduction, resulting in enhanced cancer growth, invasion and metastasis. Although epigenetic therapy has a rational and profound basis in theory, some problems remain to be discussed and solved. The first and most important is the problem of selectivity. Epigenetic events are ubiquitously distributed across normal and cancer cells. In fact, some cancers depend on certain epigenetic alterations and can be sensitive to this regulation, whereas under usual regulation, normal cells have the ability to compensate for these epigenetic changes. Therefore, the priority is to determine the most important epigenetic alterations for different cancers. The second problem extends from the first problem. Thus far, epigenetic therapy has obtained impressive results in hematological malignancies but not in solid tumors. The properties of hematological malignant cells and solid tumor cells are different. However, researchers have still investigated the appropriate strategies for solid tumors. Since epigenetic alterations have effects on the sensitivity of small molecule targeted therapy and chemotherapy or radiotherapy, epigenetic-targeted therapy seems to be an important adjunctive therapy. The combination of epigenetic therapy and immunotherapy has also been investigated in preclinical and clinical trials.

Based on the achievements obtained, epigenetic-targeted therapy is a promising strategy for anticancer treatment. Epigenomes in cancer are related to many aspects during cancer initiation. A better understanding of the specific mechanisms underlying those alterations in different cancers is necessary. Meanwhile, optimized treatment options, including a variety of combinations, still remain to be discovered.
